# What was the role of palaeogeography in East Asian monsoon evolution since the Cretaceous?

**DOI:** 10.1093/nsr/nwag534

**Published:** 2026-09-04

**Authors:** Alex Farnsworth, Daniel J Lunt, Paul J Valdes, Robert A Spicer, Stuart Robinson, Lin Ding, Zhongyu Xiong, Caitlyn Witkowski, Christopher R Scotese, Paul Markwick, Richard D Pancost

**Affiliations:** School of Geographical Sciences and Cabot Institute for the Environment, University of Bristol, Bristol BS81SS, UK; State Key Laboratory of Tibetan Plateau Earth System, Environment and Resources (TPESER), Institute of Tibetan Plateau Research, Chinese Academy of Sciences, Beijing 100101, China; School of Geographical Sciences and Cabot Institute for the Environment, University of Bristol, Bristol BS81SS, UK; School of Geographical Sciences and Cabot Institute for the Environment, University of Bristol, Bristol BS81SS, UK; School of Environment, Earth and Ecosystem Sciences, The Open University, Milton Keynes MK7 6AA, UK; School of Geography, South China Normal University, Guangzhou 510631, China; State Key Laboratory of Biocontrol and Guangdong Key Laboratory of Plant Resources, School of Life Sciences/School of Ecology, Sun Yat-sen University, Guangzhou 510275, China; Applied Research Center for Tropical Plant Conservation, Xishuangbanna Tropical Botanical Garden, Chinese Academy of Sciences, Mengla 666303, China; Department of Earth Sciences, University of Oxford, Oxford OX1 3AN, UK; State Key Laboratory of Tibetan Plateau Earth System, Environment and Resources (TPESER), Institute of Tibetan Plateau Research, Chinese Academy of Sciences, Beijing 100101, China; State Key Laboratory of Tibetan Plateau Earth System, Environment and Resources (TPESER), Institute of Tibetan Plateau Research, Chinese Academy of Sciences, Beijing 100101, China; School of Earth Sciences, and Cabot Institute, University of Bristol, Bristol BS8 1TS, UK; Organic Geochemistry Unit, School of Chemistry, University of Bristol, Bristol BS8 1QU, UK; Department of Earth and Planetary Sciences, Northwestern University, Evanston, IL 60208, USA; Knowing Earth Limited, Otley LS21 3JP, UK; School of Earth Sciences, and Cabot Institute, University of Bristol, Bristol BS8 1TS, UK; Organic Geochemistry Unit, School of Chemistry, University of Bristol, Bristol BS8 1QU, UK

**Keywords:** monsoon, Cenozoic, geology, Cretaceous, palaeoclimate

## Abstract

The evolution of the East Asian summer monsoon (EASM) has been linked to the development of the Tibetan Plateau, yet monsoon simulations vary considerably between different palaeogeographic models due to inherent uncertainties in reconstructing deep-time landscapes, both locally, regionally and globally. Using the fully coupled HadCM3BL–M2.1aD palaeoclimate model, we examine EASM evolution over the past 145 Myr across three independent palaeogeographies (Getech, Scotese and Robertsons). While all simulations capture broad hydrological trends, they diverge markedly in the timing and intensity of monsoon development. The Cenozoic strengthening of the EASM aligns with Tibetan and regional mountain orogeny, but Cretaceous behaviour reflects strong remote teleconnections driven by continental configuration. These results demonstrate that Asian monsoon systems are sensitive not only to local orography but also to global palaeogeographic context due to the impact of teleconnections on the EASM. Constraining such uncertainties is essential for resolving the monsoon’s long-term evolution and climatic influence.

## INTRODUCTION

Over the last 50 years the evolution of the East Asian summer monsoon (EASM) has been widely debated due to the diversity of inferences based on a wide range of geological proxies, few of which yield definitive monsoon signatures, and a plethora of differently configured climate models. The earliest studies suggested that monsoon inception first occurred during the Pliocene (2.5–5.3 Ma) [1–3], but subsequent records have been interpreted to provide evidence that monsoonal conditions may have existed further back in time, including during the Miocene (5.3–23 Ma) [4–7], and later, the Oligocene (23–33.9 Ma) [8,9]. More recently, monsoonal conditions have been proposed for the Eocene (33.9–56 Ma) based on both models and proxies [10–13], and some suggest it may even have existed as far back as the beginning of the Cretaceous (∼145 Ma) [11,14], although not throughout what is considered the entire modern monsoon region. So great is the literature concerning the evolution of the East Asian monsoons we cannot review all of it here, but to try and make sense of the wealth of the often-conflicting proxy and modelling arguments we explore the impact of three widely used and equally plausible palaeogeographic reconstructions throughout the variously proposed periods of EASM evolution using a single climate model. First, though, we consider the features of the EASM.

The EASM, a component of the Asian monsoon (AM) system, is a fundamental part of the global climate system, transferring atmospheric energy, momentum, moisture and mass around the world. Understanding EASM dynamics is important due to the impact it has on food and water security throughout Asia where the lives and livelihoods of over 3 billion people, including much of the global economy, are supported by the periodic rains. Furthermore, the EASM has global-scale impacts through teleconnections [15–18]. A teleconnection is a persistent pattern of weather/climate that is transmitted through the atmosphere to other parts of the planet. For example, Tibetan Plateau heating generates stationary Rossby waves that enhance subtropical westerly jet streams (and to a lesser extent the polar jet) in the northwestern Pacific, meaning that the Tibetan Plateau impacts North American climate [19–21]. The teleconnections of an enhanced EASM can likewise amplify atmospheric wave trains across Eurasia, affecting downstream temperature and precipitation patterns over Europe [22]. Teleconnection from an enhanced EASM season can also lead to greater northward heat transport to the higher latitudes, resulting in reduced sea ice extent, especially through modulation of the polar vortex [23]. Understanding the mechanisms underpinning EASM dynamics are therefore critical to defining their global impacts and predicting future EASM behaviour.

Key mechanisms responsible for the EASM on geological timescales include both internal and external forcing. Today, both internal variability and external forcings (primarily anthropogenic atmospheric CO_2_, CH_4_ and aerosol concentrations) influence energy imbalances in the climate system, driving climate variability across different timescales (e.g. inter-annual to inter-decadal). Over longer timescales (10^3^–10^4^ years) orbital forcing (variations in eccentricity, obliquity and precession) will also lead to changes in the seasonal and spatial distribution of incoming solar radiation, which in turn gives rise to variations in the monsoon seasonal length, peak intensity and spatial extent of the EASM [24]. Orbital forcing modulates the EASM primarily through precession-driven changes in boreal summer insolation, which alter land–sea thermal contrast, moisture transport and monsoon precipitation, while obliquity and eccentricity influence the system indirectly by modifying meridional temperature gradients, large-scale circulation (atmospheric jets, fronts), ice-volume feedbacks and the amplitude of glacial–interglacial hydroclimate variability. It has been argued that sensitivities to orbital forcing in warmer climates of the past may explain the inferred wet–dry fluctuations of the arid belt that persisted for much of the Paleogene in East China, reconciling contradictory proxy records [25–27].

The internal forcings on the EASM revolve largely around differential heating, e.g. land–ocean thermal contrast [28], thermal forcing over the Tibetan Plateau [11,29], Eurasian snow cover [30], vegetation feedbacks and soil moisture availability [31]. External forcings on the EASM, on the other hand, revolve largely around orbital variability and indirectly around diverse heating phenomena, e.g. El Niño–Southern Oscillation [32]; the Indian Ocean Dipole [33]; Pacific Decadal Oscillation [34]; increasing greenhouse gases [35] and associated warming variations in atmospheric aerosols [36]; and Quasi-Biennial Oscillation [37].

Many of the internal and external forcings today are, however, not the key drivers over geologic timespans. Instead, the role of palaeogeography and orography have been shown to dominate the behaviour of the EASM [11,12,38–43], although atmospheric CO_2_ has also been suggested to play a primary role [42,44,45]. However, a previous series of numerical simulations covering 0–145 Ma showed that a doubling of *p*CO_2_ only led to a −1% to +13% change in EASM precipitation [11], an outcome that has been subsequently confirmed by other modelling studies [46,47].

The influence of the development of the Pan-Tibetan Highlands (PTH) [48]—defined as the area encompassing the broader Tibetan Plateau, the Himalaya, the Hengduan Mountains and the Mountains of Central Asia (Hindu Kush, Karakoram and Tian Shan)—on the EASM over geologic time scales has been extensively investigated. Proxy evidence and palaeoclimate model simulations have suggested that the behaviour and development of the EASM is primarily determined by the evolving topography of the PTH [7,11,12,40,41,43,49–51]. How the PTH orography developed has been debated actively, beginning with the suggestion the whole Tibetan Plateau region underwent rapid uplift as one block during the late-to-mid-Miocene (∼10 Ma) [52–54]. More recently, oxygen isotope palaeoaltimetry was used to suggest that an elevated Tibet, a ‘proto-plateau’ configuration, already existed by the mid-to-Late Eocene (approximately 35 to 45 Ma) with an average height of ∼4 km [55–57]. However, within the last decade a more complex picture has emerged with extensive elevated topography present in the region since at least the Late Cretaceous in the form of a 4–5 km high Gangdese Arc upland along the southern margin of the PTH (preceding the rise of the Himalaya), while to the north another east–west trending mountain range known as the Qiangtang Highlands (the modern Tanggula Mountains), also at 4–5 km, bounded a Central Tibetan Valley (CTV) [58–62]. Beginning in eastern Tibet, the floor of the CTV progressively rose through the Eocene to reach near modern-day elevation (∼4 to 4.5 km) by the Oligocene [60–63], with the combined effect leading to a near Modern-like plateau by the start of the Neogene. Subsequently, the Himalaya surpassed the height of the Gangdese Mountains at around 15 Ma (mid Miocene) [64].

While the role of the PTH in determining EASM characteristics has been shown to be important, teleconnections linked to more far-reaching changes in palaeogeography (distant mountains, ocean gateways, ice sheet extent) could also play a role. Changes in regional palaeogeography, such as the rise of the Himalaya [64–67], closure of the Paratethys [49,68–70], uplift of the Iranian plateau/Zagros mountains and development of the East African highlands [11,38,41] likely also influenced the evolution of the EASM system. However, each different palaeogeographic realisation, although using very similar techniques to reconstruct past landforms, uses a range of different ‘first principle’ assumptions (e.g. plate convergence and divergence rates, polar wander, weathering rates, volcanic hot spot activity, etc.). Proxy-evidence, where available, may then be used to evaluate and constrain each reconstruction, although in some workflows ‘first principle’ approaches take precedence. While the inclusion of proxy-evidence (e.g. stratigraphy, sedimentology, palaeoaltimetry data, etc. and their interpretation of the geodynamic state (compression, extension), as well as their depositional setting) to constrain a reconstruction is undoubtedly of benefit. There are also inherent uncertainties and assumptions associated with their incorporation since a lot of older palaeoaltimetry is now known to be unreliable [71,72]. Different reconstruction techniques can lead to notably dissimilar realisations for the same time period.

There are numerous different palaeogeographic reconstructions and all cannot be evaluated for their impacts on climate simulations. Here, we explore the differences between three widely used but distinct palaeogeographic time series (Getech, Scotese and Robertsons; Figs S1–S3 and see Materials and methods section for further details) with highly dissimilar mountain heights, gateway configurations and land ice sheet extents. While the land–sea distribution (a function of the assumed sea level as well as the underlying palaeogeography) is generally similar, even small changes in evolving global palaeogeographies can theoretically lead to dissimilar teleconnected impacts on, and from, the EASM. Broadly, Getech has greater and more extensive relief, representing the upper limit of reconstructed topography compared to Robertsons and Scotese. The Scotese reconstructions exhibit the least relief throughout the 0–145 Ma period.

For the EASM region, the largest differences are in how the evolution of the PTH topography is represented. Each series has highly dissimilar reconstructions of Cretaceous topography, with the exception of the Early Cretaceous. The Early Cretaceous exhibited the lowest topographies across the PTH (1000–1500 m; 25–30°N) throughout the last 145 Myr, but Getech does have an SW–NE trending mountain range (∼3000 m; ∼30°N) that is not present in Scotese or Robertsons. Generally, low topographies in Asia persist until the late Eocene in both Scotese and Robertsons, while significant increases in elevation starts in the mid-Cretaceous in Getech. Cretaceous elevations are currently highly unconstrained. By the late Eocene, all three series start to converge with high elevations in the PTH as a result of Indian–Asian plate collisional forces.

Although some prior work has hinted that palaeogeography, and in particular palaeotopography, may play a role in determining the existence and dynamics of monsoon climates in the EASM region [12,38,41,43,47,57]; this has not been tested systematically throughout the time when the EASM is most likely to have evolved. To understand the possible spatiotemporal impact of these three different palaeogeographies (Getech, Scotese and Robertsons) on potential monsoon conditions within the EASM region through the last 145 Ma, we use the HadCM3L palaeoclimate model. *p*CO_2_ levels for each time slice were set by proxy data (Getech and Scotese) or by using idealised values (Robertsons).

## RESULTS

**Figure 1.**
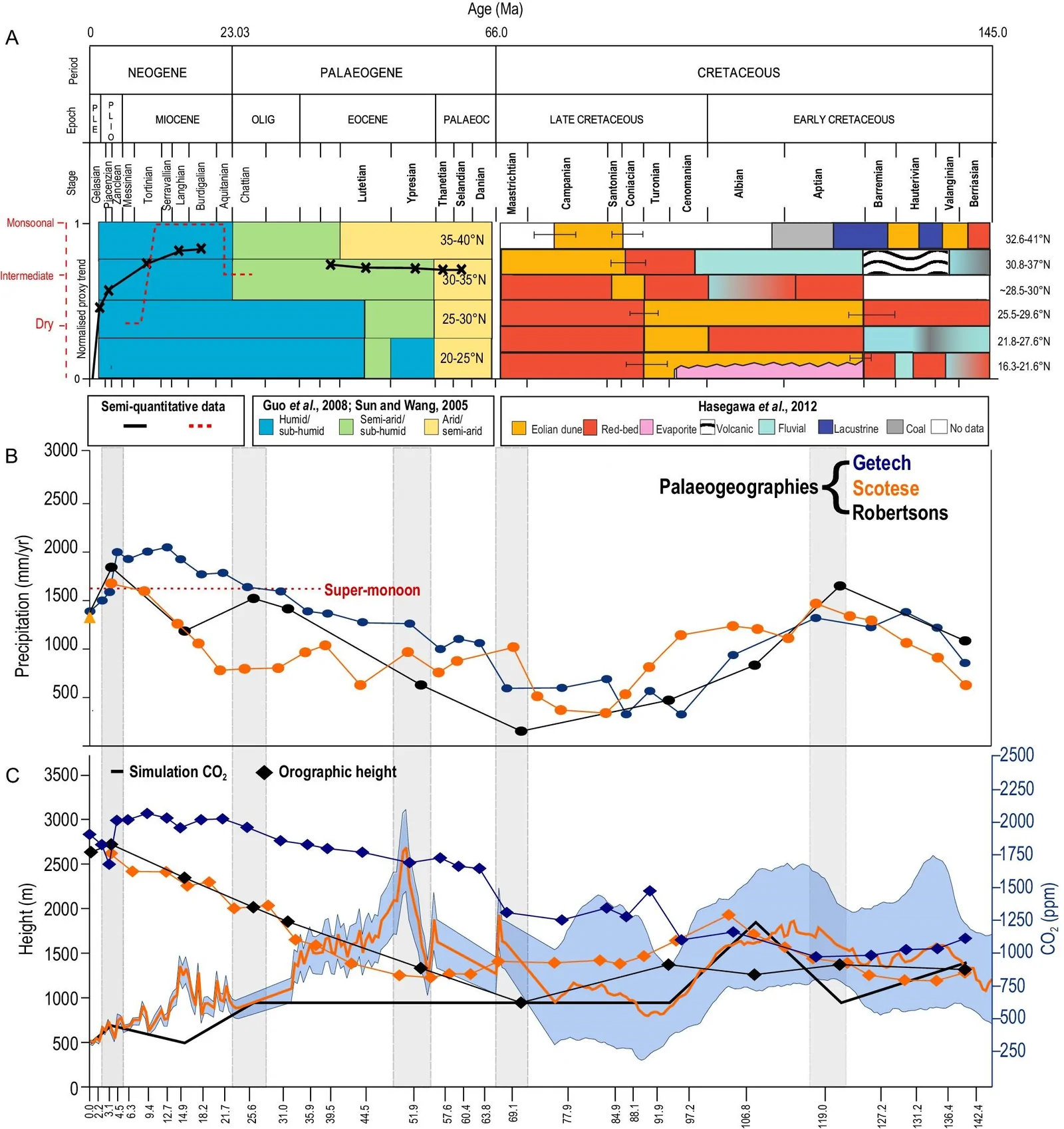
East Asian (EA) precipitation evolution through time. (A) Normalised quantitative precipitation proxy data trend (solid black line) is shown in conjunction with two qualitative proxy data compilations for the Cretaceous (Hasegawa *et al.* [73]) and for the Paleogene and Neogene (Guo *et al.* [75]; Sun and Wang [4]) within 16°N–41°N, 75°E–130°E. Both coloured panels in (A) are independent of either of the left *y* axis and indicate the intensity of the hydrologic cycle. The dashed red line (Clift *et al.* [94]) is a compilation of proxies from ocean drilling program sites 1146 and 1148 indicating monsoonal conditions. See the Supplementary Materials in Farnsworth *et al.* [11] for details. (B) EA modelled mean annual precipitation (mm/yr) for each geologic stage for three independent palaeogeographic series: (i) Getech (blue line), (ii) Scotese (orange line) and (iii) Robertsons (green line). All palaeogeographies are shown in Figs S1–S3. Orange triangle represents the mean annual (1979–2011) precipitation for the monsoon region from CMAP observations. Red stippled line indicates super-monsoon conditions (where the annual mean precipitation is >*P* + *P*2σ, where *P* is the pre-industrial 30-year mean of precipitation and *P*2σ is two standard deviations of the interannual variability of the pre-industrial precipitation). (C) Mean orographic height (m) between 10°N and 45°N, 60°E and 110°E (masked > 1000 m) for each palaeogeographic series geologic stage (diamonds) and CO_2_ concentrations (line) for (i) Getech (blue), (ii) Scotese (orange) and (iii) Robertsons (green). Shaded blue band signifies range in proxy CO_2_ uncertainty (Foster *et al.* [99]; Rae *et al.* [82]). PALEAOC, Palaeocene epoch; OLIG, Oligocene epoch; and PLE, Pleistocene epoch. Grey vertical bars indicate time periods of interest.

The three palaeogeographic series (Getech, Scotese and Robertsons) show broadly consistent hydrological responses (annual precipitation) across the EASM region from the Cretaceous to the Modern (Fig. 1b). Annual precipitation for each series shows the Early Cretaceous was humid to very-humid, which is supported by observations on climatically sensitive sediments (e.g. lacustrine and fluvial deposits) for the East Asian monsoon region [73]. During the Early Cretaceous (Barremian–Valanginian), all three reconstructions show near observed (CPC Merged Analysis of Precipitation (CMAP); 1979–2009) Modern day values in annual precipitation (Fig. S17). A similar response using the Scotese palaeogeography using a different palaeoclimate model (CESM1.2) also showed agreement with monsoonal conditions during the early Cretaceous [47].

All three of the palaeogeographic series show aridification in the EASM region by the mid-Cretaceous (Albian–Turonian), consistent with proxy-evidence (Fig. 1a and b). While the Scotese series also displays this aridification, humid conditions (>1000 mm/year) persist until ∼90 Ma, which is inconsistent with the demonstrable presence of widespread aeolian dune, red bed and evaporite sedimentological evidence [73]. Broadly, from ∼100 to 50 Ma each palaeogeographic series simulates annual precipitation for the EASM region that is typical of the Mediterranean today (>900 mm/year), but each palaeogeography shows small variations in persistence. Precipitation intensification begins during the Early-to-mid Paleogene for Getech and Robertsons (∼63 Ma), in agreement with proxy-evidence (Fig. 1a), while the Scotese series shows a much later (Early Miocene; ∼22 Ma) precipitation increase for the EASM. The Getech series shows a greater increase in annual precipitation compared to both the Scotese and Robertsons during all periods of the Cenozoic. In particular, Getech produces ‘super-monsoon’ [11] conditions during the Miocene. This is defined as when the annual mean precipitation is >*P* + *P*2σ, where *P* is the pre-industrial (PI) 30-year mean of precipitation and *P*2σ is two standard deviations of the interannual variability of the PI precipitation. Both Robertsons and Scotese do not show similar ‘Super-monsoon’ conditions being established. Model output interrogation shows the increased monsoon response in Getech, when EASM precipitation is greater than today, is likely due to a higher and broader Tibetan Plateau (Fig. 1c; Figs S1–S3) leading to strengthened thermal forcing (integrated column heating; Figs S27–S29) in the mid-troposphere. This strengthens low pressure near the surface and high pressure in the upper troposphere. In turn, this amplifies the land–ocean temperature gradient, promoting increased moisture transport into East Asia. A higher and broader Tibetan plateau (and Zagros Mts/Iranian plateau [11,41]) in Getech also impacts mechanical forcing by blocking and steering greater moisture along low-level westerlies into the EASM region (Figs S5–S7). Further afield, palaeogeographic differences (e.g. other mountain ranges, Paratethys, continental configurations and/or gateways) may also be acting to modify EASM precipitation differences. Future sensitivity analysis is needed to quantify this conclusively.

**Figure 2.**
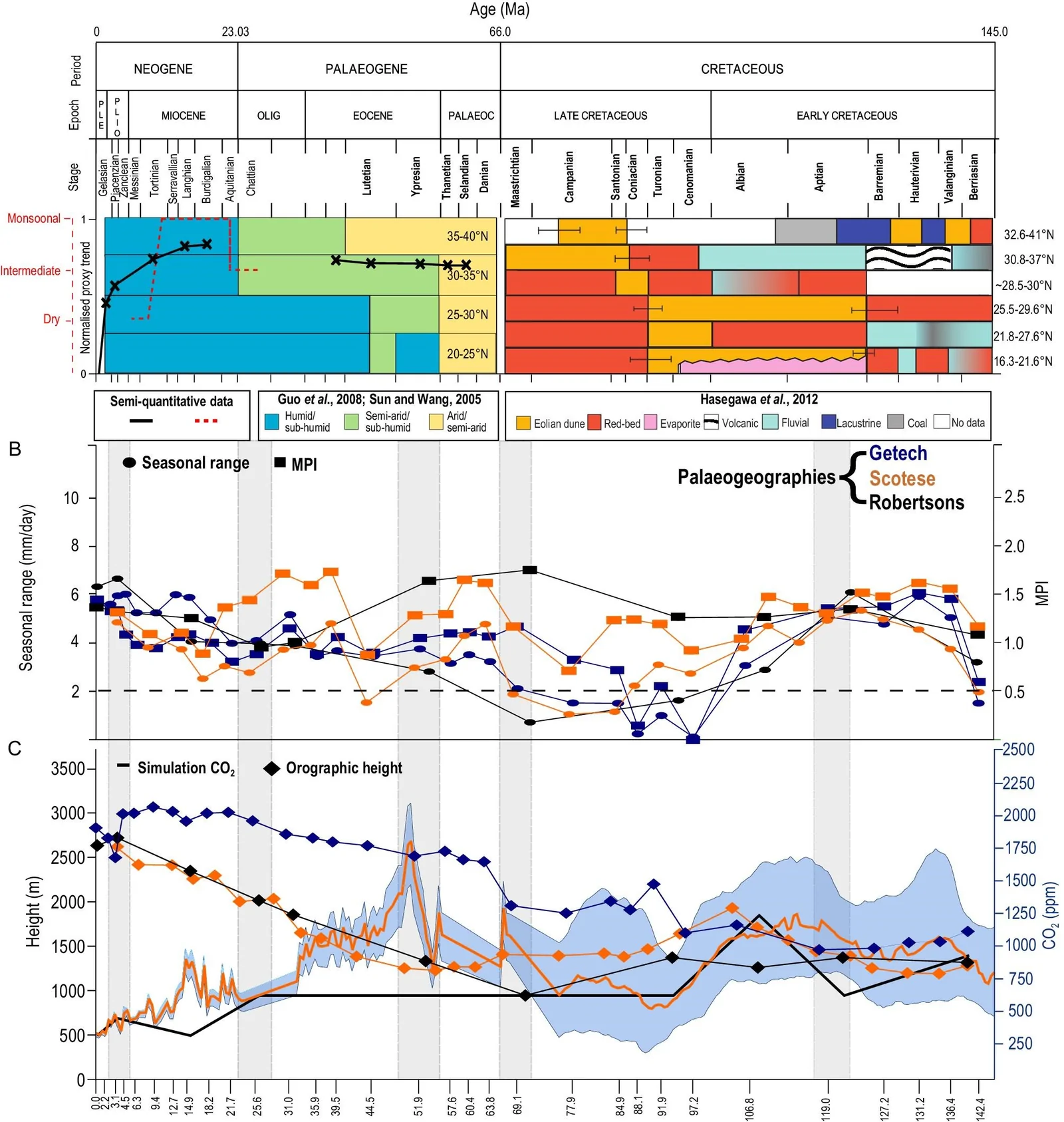
East Asian summer monsoon indices through time. As Fig. 1, but (B) circles depict the seasonal range (May–September minus November–March precipitation) and squares represent MPI (May–September minus November–March precipitation divided against annual precipitation) for (i) Getech (blue), (ii) Scotese (orange) and (iii) Robertsons (green).

In each of the palaeogeographic reconstructions, the Cenozoic increase in annual precipitation is highly correlated with the timing of significant mountain and plateau building across the PTH, but this is not the case during the Cretaceous. While climatological annual precipitation provides a plausible explanation of occurrences of climatically sensitive sediments (e.g. gypsum, jarosite, red beds, coal) and palaeovegetation (e.g. revealed by pollen and megafossils), annual precipitation itself is no measure of monsoon conditions. Ideally, seasonally resolved proxies would provide a more robust comparator, however, their spatiotemporal distribution in deep time is limited [74]. Instead, we use two model-based monsoonal climate indicators to assess how active the EASM was for each palaeogeographic reconstruction. The modern seasonal range of precipitation (defined as: May–September minus November–March precipitation) and Monsoon Precipitation Index (MPI; May–September minus November–March precipitation divided against annual precipitation) indicates that monsoonal conditions may have been present in some parts of the EASM region since at least ∼145 Ma (Fig. 2b). This is predominantly on the coasts of China and southerly latitudes at the extremities of the EASM region likely to have been exposed to seasonal precipitation generated by migrations of the Inter-Tropical Convergence Zone.

Getech simulations for the middle-to-Late Cretaceous indicate that monsoon conditions cease in the EASM region with a MPI > 0.5 and a seasonal range > 2 mm/day, before becoming re-established near the end of the Cretaceous. On the other hand, although both Scotese and Robertsons reconstructions predict Late Cretaceous aridification, they are still considered monsoonal in terms of their MPI > 0.5, although the seasonal range does also decrease to 2 mm/day indicating a more complex signal. Indeed, the Scotese palaeogeographic MPI response suggests strong monsoonal conditions were present across much of the EASM region throughout the last 145 Myr at near modern values. Even though most proxies cannot supply a definitive monsoon signature, they show considerable variations in overall hydroclimate and no monsoon continuity [73,75].

More strikingly, from the mid-to-Late Cretaceous to Oligocene there is little overall agreement among the palaeogeographies in terms of MPI and seasonal range, indicating that the dynamic responses of the hydrologic cycle are being promoted by different process and driving mechanisms, likely driven by differences in local, regional and global palaeotopography. For each palaeogeographic series, Cenozoic mountain and plateau building (Figs S1–S3) produces a larger seasonal range in precipitation and MPI (Fig. 2b). During the Cretaceous, changes in the seasonal range of precipitation and MPI are not associated with changes in the topography of the PTH region (Fig. 2b and c). The different responses of the palaeogeographies in Figs 1b and 2b suggest that other non-CO_2_ and PTH factors are having an impact on the gross moisture flux into East Asia, likely driven by teleconnections external to the PTH region. Modern–palaeo comparisons of EASM seasonality indicate that when the monsoon in the EASM region was stronger than today in the Cenozoic for the Getech reconstruction, both the wettest-month peak precipitation and Spring precipitation increased along with increase in dry season precipitation (Fig. S17), in agreement with He *et al.* [76]. This trend was not evident in Scotese or Robertsons reconstructions. Between each palaeogeography, the spatial distribution of precipitation varies both in the summer (wet) (March–September), as well as during the winter (dry) (December–February), even when the annual signal is similar (Fig. 1b). Summer precipitation distribution for each time period is shown for June–August (Figs S5–S7). For brevity, here, we investigate four key time periods, the Aptian, Maastrichtian, Chattian and Gelasian, when significant changes in the EASM have been observed both in multiple proxies and simulations.

### Aptian (121.4-113.2 Ma)

Both proxy and model evidence indicate that the Early Cretaceous experienced pronounced monsoonal conditions (MPI 1.2–1.5), shown in the models to be summer-wet. During the Aptian, identified as the wettest interval of the Early Cretaceous, all three palaeogeographic reconstructions suggest that a monsoon over Asia reached its greatest intensity, with annual precipitation comparable to modern values (Figs 1b and 2b). Despite notable differences in topographic representation, with Getech featuring more extensive mountainous regions (∼2000 to 2500 m) and Scotese depicting relatively subdued relief, all three reconstructions yield broadly consistent annual and seasonal precipitation totals.

**Figure 3.**
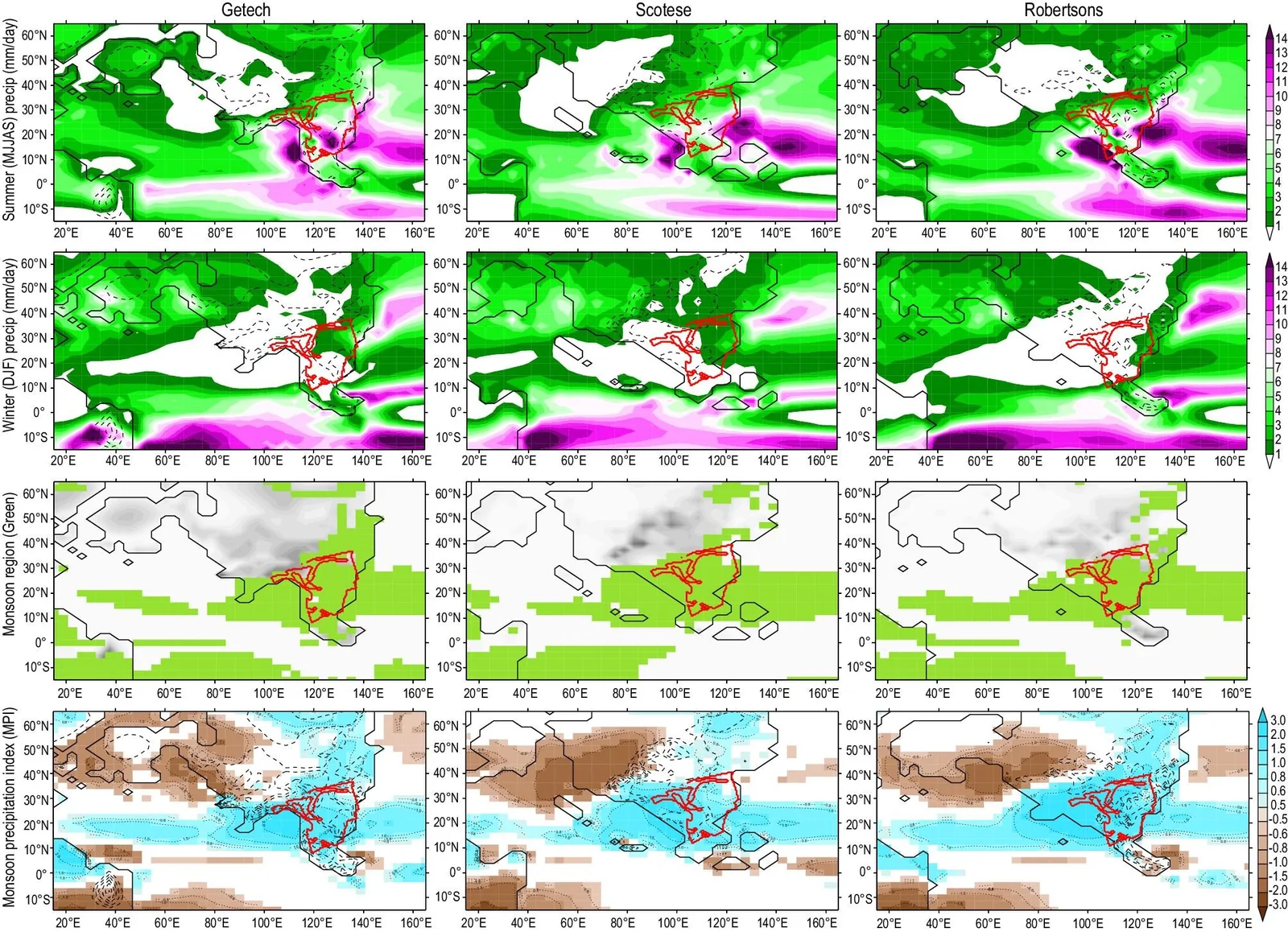
Aptian (121.4-113.2 Ma) monsoon statistics. Summer May–September (MJJAS) precipitation (mm/day), Winter December–February (DJF), Monsoon Domain (green area) and MPI for (i) Getech, (ii) Scotese (Early Aptian) and (iii) Robertsons palaeogeographic series. Red region designates the modern rotated East Asian monsoon region.

The spatial distribution of precipitation (Fig. 3) exhibits maxima across low latitudes (10–25°N), decreasing poleward. In the Scotese reconstruction, enhanced moisture penetration extends into the Asian continental interior (∼90°E), primarily through recycled precipitation, whereas in both the Getech and Robertsons reconstructions, rainfall remains largely confined to eastern Asia (>100–110°E). Precipitation intensity is greatest in the Getech simulation, with less in Robertsons, reflecting the influence of elevated topography that enhances orographic uplift and slope heating, thereby intensifying onshore flow and producing a pronounced rain-shadow effect.

Winter precipitation patterns (Fig. 3) show stronger agreement among the reconstructions, with all indicating limited rainfall across the modern EASM region and in the continental interior, except along coastal margins where light precipitation (1–3 mm/day) occurs. For each reconstruction this overall pattern is due to dry–cool westerlies persisting over much of the Asian continent (Figs S21–S23) throughout the Cretaceous. Uniquely, the Scotese reconstruction shows light precipitation (∼1 mm/day) extending farther inland during winter months.

All three reconstructions exhibit broadly comparable monsoon intensities (MPI > 1.0) and similar Monsoon Domains (MDs; Wang and Ding [77]) defined as >55% of annual precipitation falling during local summer (May–September) and the local summer-minus-winter precipitation rate being >2 mm/day. However, the processes governing monsoon precipitation differ among the reconstructions. The maximum latitudinal extent of the ascending branch of the Hadley circulation (Figs S14–S16) reaches ∼15°N during the Aptian summer in all cases, coinciding with the latitude of maximum summer precipitation. In the Getech reconstruction, however, the Hadley cell is weaker than in both Scotese and Robertsons, whereas Scotese displays a more equatorward contraction of the Hadley cell relative to the other two.

All three reconstructions also indicate enhanced convective activity poleward of the inter-tropical convergence zone (ITCZ) (Figs S14–S16), suggesting that a secondary ‘sea-breeze’ monsoon circulation operated concurrently, being most pronounced in the Scotese reconstruction. This is determined using atmospheric streamfunction, a measure of atmospheric convergence and vertical wind velocities that show the location of the main ascending regions associated with the ITCZ. The strength and latitudinal extent of this sea-breeze circulation varies between each palaeogeography. Although the ITCZ is comparatively weaker in the Getech simulation, EASM-region Moist Static Energy (MSE; Fig. S18 xxvi) maxima (a measure of the boundary of moist convergence) occur farther poleward of the ITCZ in Getech (∼35°N; Fig. S19 xxv) than in either Scotese (∼20°N; Fig. S18) or Robertsons (∼20°N; Fig. S20 ix), implying that sea-breeze-driven circulation and topographic moisture capture in the south dominated the monsoon signal, rather than a more poleward migration of the ITCZ itself [78]. The penetration of this ‘sea-breeze’ is variable between each palaeogeography (Fig. 3). Future work should centre on a full set of sensitivity studies for each reconstruction to trace the physical mechanism for the response of the Hadley and sea-breeze circulation by varying local and regional palaeogeography.

### Maastrichtian (72.2-66.0 Ma)

**Figure 4.**
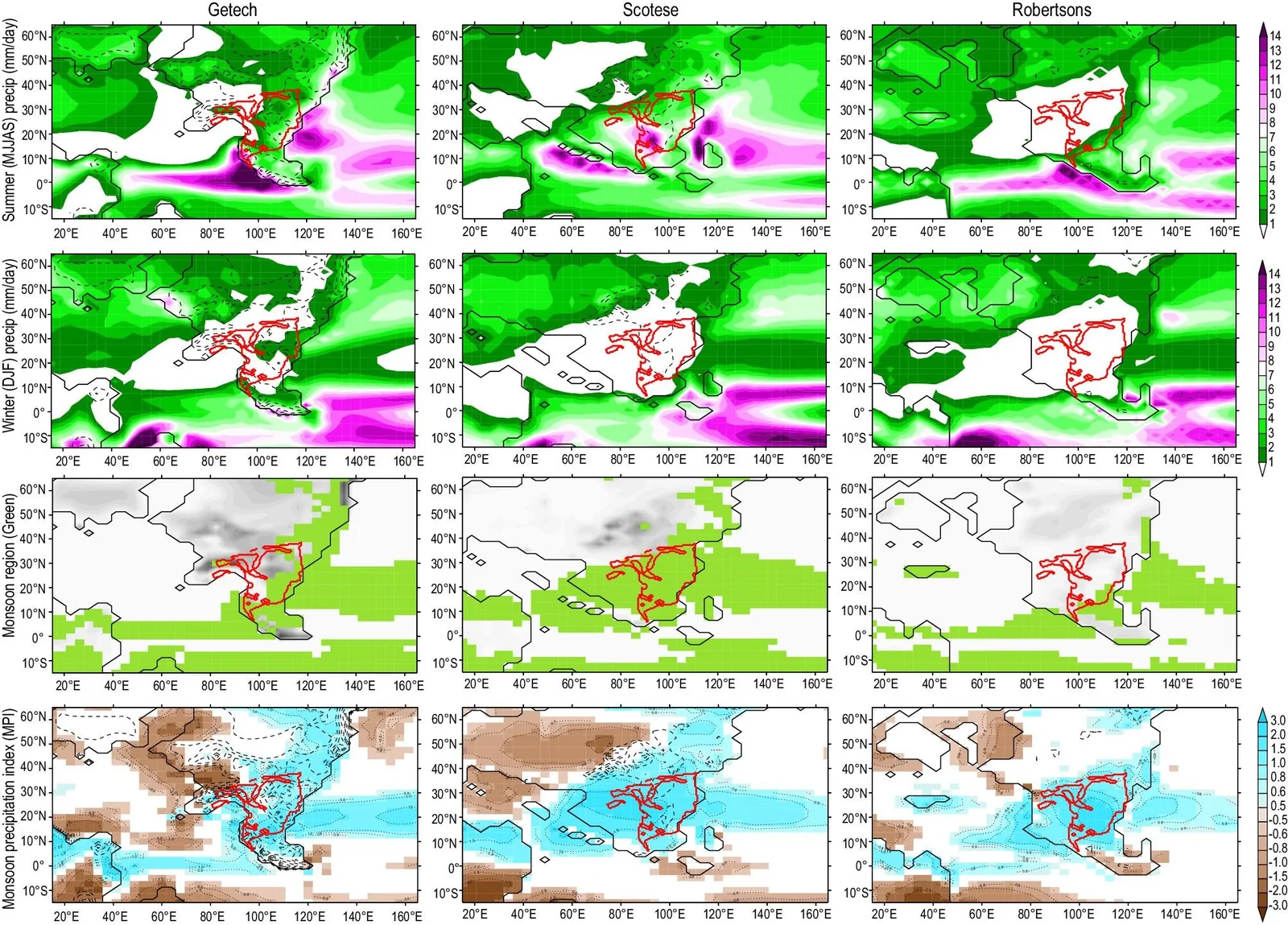
Maastrichtian (72.2-66.0 Ma) monsoon statistics. Summer May–September (MJJAS) precipitation (mm/day), Winter December–February (DJF), Monsoon Domain (green area) and MPI for (i) Getech, (ii) Scotese and (iii) Robertsons palaeogeographic series. Red region designates the modern rotated East Asian monsoon region.

Across reconstructions there is agreement that Maastrichtian (72.2-66.0 Ma) summer precipitation was substantially lower than in the Aptian. However, the magnitudes and spatial patterns differ: the Scotese reconstruction yields markedly higher summer precipitation across the EASM region, whereas Getech and Robertsons simulate little to no summer precipitation (Fig. 4). This contrast is not explained by systematically higher coastal orography in Getech, as in the Aptian, when precipitation totals approach modern values. Instead, the suppression of Maastrichtian EASM region rainfall in Getech and Robertsons likely reflects remote controls (e.g. gateway states, stationary-wave adjustments or cryosphere influences) that modulate moisture transport and ascent over East Asia.

Winter precipitation is minimal in all three reconstructions over the EASM region as well as in the Asian continental interior (Fig. 4). This is due to dry, cool low-level westerlies dominating most of the continent (Figs S21–S23). Both Getech and Robertsons, therefore, indicate the absence of a Maastrichtian monsoon, with no MD simulated in Robertsons and only a narrow coastal MD in Getech. In contrast, Scotese produces a broad MD extending well into the continental interior (Fig. 4) resulting from moist, warm, smooth inflow though most of the Cretaceous (Fig. S22).

Despite these differences in MD extent, MPI values are high in all three reconstructions. This should not be interpreted as evidence for a robust monsoon. Rather, it is an artefact of very low absolute precipitation in summer coupled with even lower winter totals, which inflates the seasonal contrast in MPI. In other words, high MPI under Maastrichtian aridity reflects relative seasonality, not the presence of a dynamically organised monsoon circulation.

### Ypresian (56.0-48.1 Ma)

The Early Eocene (Ypresian; 56.0-48.1 Ma) shows the greatest divergence in annual precipitation response among the three palaeogeographical reconstructions. Robertsons (∼1.6 mm/day) is the driest of the three, followed by Scotese (∼1.9 mm/day), with Getech having significantly wetter values (∼3.6 mm/day).

**Figure 5.**
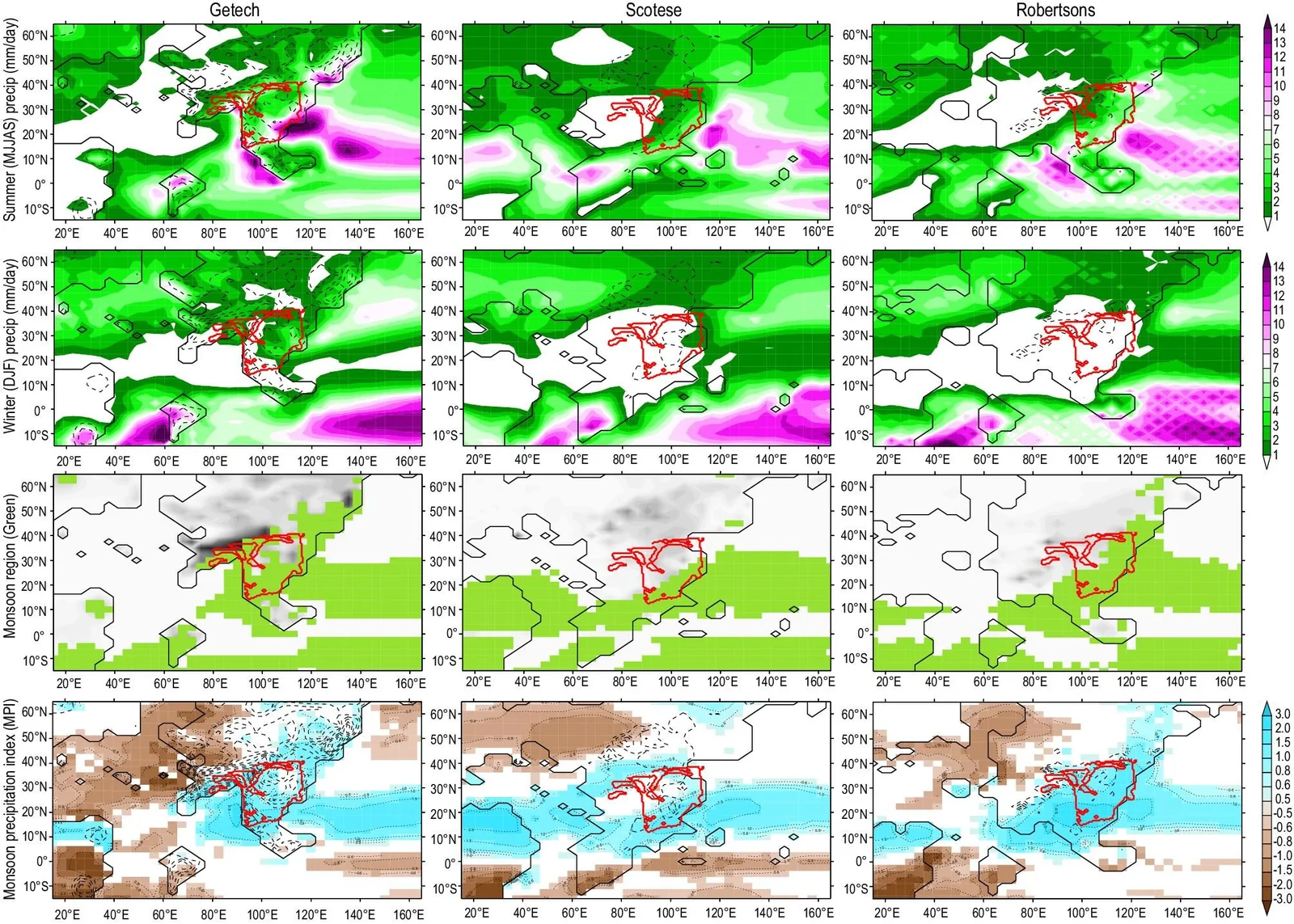
Ypresian (56.0-48.1 Ma) monsoon statistics. Summer May–September (MJJAS) precipitation (mm/day), Winter December–February (DJF), Monsoon Domain (green area) and MPI for (i) Getech, (ii) Scotese and (iii) Robertsons palaeogeographic series. Red region designates the modern rotated East Asian monsoon region.

All three palaeogeographies show a similar precipitation distribution during the monsoon season where a demarcation between wet (within the East Asian) and dry (continental interior) regions is aligned along a southwest-northeast (Fig. 5) boundary. Moisture penetration into the continental interior is greater in Scotese (extending to ∼90°E), with both Getech and Robertsons showing limited moisture penetration (∼100°E). However, in Getech and Scotese any moisture that does penetrate continental interiors is small (1–3 mm/day).

During the winter, both Scotese and Robertsons display little to no precipitation as a result of cold, dry westerlies prevailing across the mid-to-high latitudes of the Asian continent (Figs S22 and S23). However, Getech shows slightly wetter conditions (∼25°N) around the southern coasts due to southerly inflow of warmer, moist air (Fig. S21 xv). This pattern is consistent with an intensified East Asian Winter Monsoon (EAWM)-like system associated with uplift of the northern and northeastern Tibetan which modifies flow from the Siberia–Mongolia high. Broadly, this is because uplift of northern Tibet deflects more of the cold, dry northerly flow from the Siberian High away from East Asia, allowing more episodic warmer, moister southerly air to penetrate farther north. The uplift of northern Tibet also promotes stronger low-level convergence and ascent, leading to overall higher winter precipitation [79]. In Scotese and Robertsons, the same mechanism does not occur until the PTH elevated topography is sufficiently developed that the Siberian High subsequently also strengthens and cool-dry air-flow from the Asian interior moves away from the East Asian monsoon region (Figs S22 and S23, and Figs S25 and S26). It should also be noted that Getech has high orography along the eastern coast (∼50°N), the Sikhote-Alin Mountain range and later the Mongolian Mts, that is less developed or not present in Scotese and Robertsons until the Late Miocene in terms of the Mongolian Mts. These topographic features should also act to strengthen the EAWM-like system by strengthening the Siberian High in Getech leading to drier winter conditions in East Asia when topography is more developed [80,81]. Yet, this does not have a discernible impact on wetter winter conditions in southern regions.

MPIs indicate that all three palaeogeographies generate similar spatial monsoonal conditions over East Asia. This is because they all show low winter precipitation of ∼1 mm/day. Scotese and Robertsons indicate stronger monsoonal conditions (MPI > 1.5) penetrating further inland compared to Getech. The MD shows deviation from MPI. Here, only Scotese suggests widespread monsoonal conditions over East Asia, while broadly in Getech, and to an even greater extent in Robertsons, the MD is confined to coastal regions (Fig. 5).

The maximum poleward location of the ITCZ through the summer monsoon season (predominately in August, Figs S14–S16) is similar in all three palaeogeographies (∼15°N). However, MSE analysis (Figs S18–S20) shows a more poleward non-ITCZ monsoon regime [78] develops in both Getech and Scotese (∼20°N and ∼25°N, respectively), explaining the greater poleward extent of precipitation.

The divergent precipitation responses among the three palaeogeographic reconstructions are likely driven by several interacting palaeogeographic factors. First, the Getech reconstruction exhibits substantially greater topographic relief, both in elevation and spatial extent, than the Scotese and Robertson reconstructions. This would enhance orographic rainout on the windward side of topographic barriers oriented perpendicular to moisture-bearing flow, while also strengthening land–ocean thermal contrasts through slope and/or plateau heating, thereby promoting stronger moist advection. Second, land–sea distributions differ among the reconstructions. Although the palaeolatitude of the Asian continent is broadly consistent, the position of India, the subaerial extent of Greater India, the width of the palaeo-Tethys ocean and the state of key ocean gateways vary substantially. In particular, unlike the Getech and Robertson reconstructions, Scotese includes a closed Turgai Strait. These differences indirectly affect monsoon precipitation in East Asia by altering regional land–sea thermal gradients and associated atmospheric and oceanic circulation patterns. More broadly, differences in global land–sea configuration may influence monsoon behaviour through their effects on atmospheric wave propagation, the Pacific subtropical high, trade-wind strength and large-scale teleconnection patterns. Consequently, both regional and global palaeogeographic differences represent non-trivial controls on the simulated monsoon precipitation response, both in the Ypresian, and to different extents, in all time periods.

The Ypresian simulations also differ in their prescribed atmospheric *p*CO_2_. Both Getech and Scotese simulations use the proxy-derived reconstruction of Rae *et al.* [82], equivalent to 1478 ppm, whereas the Robertson simulation uses an idealised *p*CO_2_ of 1000 ppm. Higher *p*CO_2_ can, in principle, intensify monsoon precipitation by increasing atmospheric moisture holding capacity and strengthening the hydrological cycle. However, although additional sensitivity experiments with reconstruction-specific *p*CO_2_ values are beyond the scope of this study, previous work suggests that *p*CO_2_ exerts a secondary control relative to palaeogeography. For example, Farnsworth *et al.* [11] showed that, under the Getech palaeogeography, doubling *p*CO_2_ from 560 to 1120 ppm increased June–September precipitation from 4.96 to 5.91 mm/day. This indicates that *p*CO_2_ differences may contribute to the simulated precipitation contrasts, but do not primarily account for the divergent responses among the palaeogeographic reconstructions. Future work should centre on a full set of sensitivity studies for each reconstruction to confirm the physical mechanism driving the mid-Eocene response.

### Chattian (27.3-23.0 Ma)

**Figure 6.**
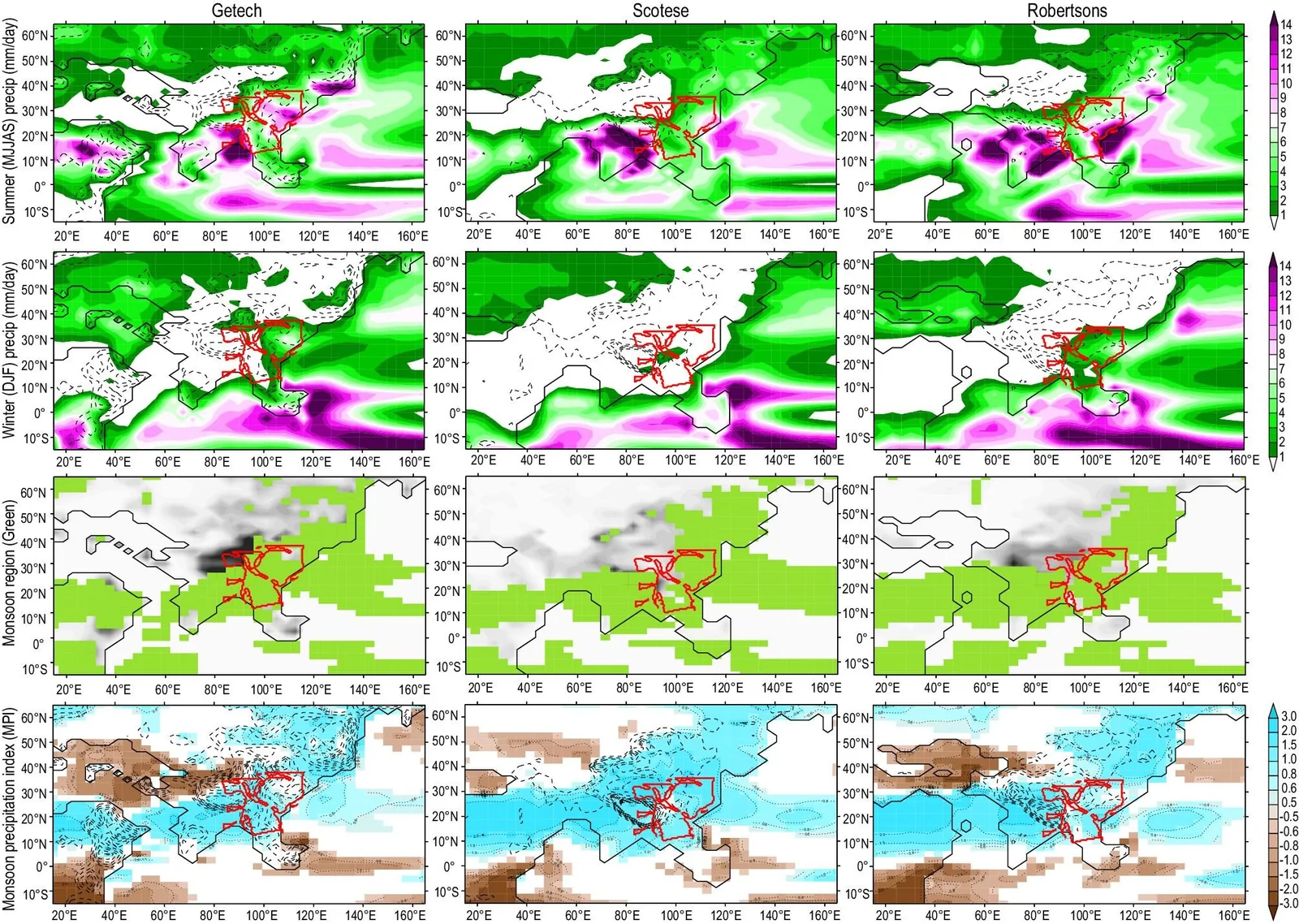
Chattian (27.3-23.0 Ma) monsoon statistics. Summer May–September (MJJAS) precipitation (mm/day), Winter December–February (DJF), Monsoon Domain (green area) and MPI for (i) Getech, (ii) Scotese and (iii) Robertsons palaeogeographic series. Red region designates the modern rotated East Asian monsoon region.

During the Chattian (27.3-23.0 Ma), all three reconstructions simulate broadly similar monsoon season precipitation patterns across the EASM region (Fig. 6). However, Scotese is drier (∼4 mm/day) than Getech and Robertsons (∼8 mm/day), yielding roughly half the seasonal total over the core EASM region. Again, moisture penetration into the continental interior is deeper in Getech and Robertsons (precipitation bands extending to ∼90°E) than in Scotese (limited to ∼100°E), indicating weaker westward inland reach in the latter (Figs S5–S7).

The Meiyu–Baiu front (Fig. 6), a well-defined extra-tropical convergence zone characteristic of the modern EASM, is present in Getech and, to a lesser extent, Robertsons, whereas Scotese lacks a comparably sharp precipitation band forming between tropical and extratropical airmasses. This contrast suggests a more coherent, dynamically organised monsoon rainband in the former two reconstructions as found today, signifying a more poleward monsoon regime rather than a more equatorward ITCZ dominated monsoon circulation [78].

In winter, Scotese remains largely dry across continental Asia, including the EASM sector (Fig. 6). Getech and Robertsons also show widespread continental aridity, but simulate enhanced winter precipitation over the EASM (∼2–3 mm day⁻¹) relative to the interior (Figs S21–S23). Similarly, as during the Ypresian, the impact of the EAWM-like dynamic that brings dry, cool air from the north is reduced due to higher blocking topography in the PTH, so allowing warmer more moist airmasses from the south to penetrate further northward (∼25°N) into East Asia [79].

Despite differences in absolute strength and seasonal range (Fig. 2b), the MD is broadly comparable among the three sets of simulations. MPI values are highest in Scotese, reflecting stronger relative seasonality under lower absolute precipitation. The average summer (JJA) ITCZ latitude over the region shows little deviation (∼10°N; Figs S14–S16), and atmospheric streamfunction diagnostics (and MSE) indicate a pronounced land–sea (sea-breeze like) summer circulation in all cases, consistent with thermomechanical forcing by Tibetan topography. A more comprehensive analysis would be required to understand the energetics of the plateau for each palaeogeography and should be a focus for future study.

### Pliocene (5.3–2.5 Ma)

By the Pliocene (5.3–2.5 Ma), the spatial distribution of precipitation in both the summer and winter seasons converges across the three reconstructions (Fig. S4). However, Robertsons shows a strong SE–NW orientated precipitation band between 20°N and 35°N compared to Getech and Scotese, which are broadly similar. This suggests a stronger Meiyu–Baiu convergence zone in Robertsons. MD and MPI likewise agree, indicating that local, regional and global palaeogeographic boundary conditions are sufficiently aligned to produce a consistent dynamical monsoon response. This is perhaps to be expected as this is the most recent timeslice.

## DISCUSSION

### Modelled EASM palaeogeographic response

Our multi-palaeogeographic reconstruction palaeoclimate simulations show that the evolutionary trajectory and absolute intensity of monsoon conditions in the EASM region are highly sensitive to palaeogeographic realisations. Differences among reconstructions, e.g. in how and when orography forms, how coastlines, bathymetry and ocean gateways evolve and how ice sheets are prescribed can produce different monsoon outcomes (intensity, ITCZ location, monsoon regime development, Meiyu–Baiu convergence, frontal system development, continental moisture penetration), including whether a given interval is comparatively monsoonal at all. To a very large extent, this explains the often contradictory outcomes of simulations to date and underscores the importance of deriving realistic palaeogeographies, particularly topographies, before meaningful discussions can be had concerning monsoon evolution. These discrepancies between palaeogeographies are particularly evident from the mid-Cretaceous through the Oligocene, when the seasonal range of precipitation and MPI diverge sharply across reconstructions. However, to quantify the individual causes of these differences requires a raft of sensitivity experiments and should be a future focus for multi-time period investigations.

Within our experiments, three components of uncertainty dominate. First, perceived topographic histories (heights, widths and spatial pattern) of the PTH differ in both magnitude and timing between the palaeogeographies, altering atmospheric circulation through mechanical and thermal forcing (both in summer and winter). Second, time sampling strategies vary (e.g. stage mid-points versus fixed 5-Myr bins versus stand-alone slices), which separates rapid tectonic and palaeogeographic transitions into different time bins making direct comparison challenging. Third, other more global boundary conditions (e.g. Antarctic ice extent, Paratethys/other gateway connectivity, shelf exposure) sometimes evolve inconsistently across palaeogeographies, affecting continental heating rates [83], which in turn lead to perturbations in stationary waves and other moisture transport pathways. These teleconnections to the EASM regions can have a first- or second-order effect on the monsoon. None of the existing reconstructions should be treated as ‘correct’; rather, each encodes specific tectonic and proxy assumptions that will have a material impact on monsoon dynamics. As such, they are hypotheses based on the interpretation of observational data, and models of elevation and former extent of environments beyond the preserved record. Reconstructing past topographic height is a fast-moving field, ever evolving with new discoveries [59,61,63,84]. It should also be noted that different palaeogeographies will also include varying amounts of proxy-data leading to their different realisations. Unfortunately, not all of the underlying data for different reconstructions are openly available, nor is it obvious how much ‘imagination’ is involved.

It is widely known that PTH evolution strongly modulates monsoon strength throughout the EASM region [11,38,41,43,85], e.g. having mountains or a plateau will enhance the monsoon compared to no topography at all. Yet our results intriguingly show that monsoon characteristics are also contingent on the broader palaeogeographic backdrop. When and where Tibetan region relief emerges changes atmospheric jets, moisture convergence and the emergence of the monsoon trough (Meiyu–Baiu frontal system); yet the mid-to-Late-Cretaceous behaviour illustrates that despite having the same CO₂ values (apart from Robertsons), each reconstruction can yield contrasting seasonal ranges and MPI depending on palaeogeographic elements external to the Tibetan region. In particular, the Maastrichtian aridity in some reconstructions versus persistent (though weak) monsoonal seasonality in others points, potentially, to remote teleconnections (e.g. Paratethys, Iranian Plateau/Zagros Mts, East African Highlands and other global mountain belts) superimposing on the regional EASM region signal. We know the rise of the East African Highlands plays an important role in the development of the Somali Jet that brings moisture into the region, yet when they rise and what critical height is required is debated [41]. Likewise, we now also know that thermal heating of the North American continent has a teleconnection on the AM [83], meaning mountain building and palaeogeographic changes there could also impact conditions in the EASM region. Such teleconnections have the potential to adjust the stationary wave field, Hadley–Walker circulations, temperature gradients, etc. shifting ascent zones and moisture flux into East Asia even when local Tibetan topography is similar.

The EAWM is also likely modulated by palaeogeographic development in all reconstructions. The timing of formation of high mountains or a plateau determines when the Siberian High develops. This occurs earlier in the Cenozoic in Getech (late Eocene), compared to Scotese (Early Miocene) or Robertsons (Middle Miocene) and leads to cool and dry winters in the EAWM region. However, the PTH can also dampen the impact of the EAWM in that it steers a portion of cool, dry airmasses away from the region if the plateau is well developed (high and broad) allowing warmer, moist airmasses from the south to penetrate further north. This weakens the winter monsoon and brings relatively wetter winter conditions in more southerly regions (∼25°N) of the monsoon region. A latitudinal component is likely, whereby more poleward plateau topography more effectively deflects or blocks cold, dry winter air from reaching the monsoon region. This view aligns with wider literature that separates thermal and mechanical monsoon forcing: plateau heating, land–sea thermal contrast and snow/soil-moisture feedbacks [11,83,86] on the one hand; barrier effects, localised jets and lee cyclogenesis (formation of a low-pressure system on the downwind side of a mountain range) on the other [11,38,85]. While both modes can operate in tandem, their relative weights are ultimately set by the palaeogeography. Put simply, an ‘only-Tibet is important’ narrative can over-simplify predictions of monsoon strength, when remote orography, gateways, etc. also play a role through configuring the background stationary waves, potentially modifying processes that drive the EASM system.

Across the full record, land–sea reorganisation from the breakup of Pangaea appears to set the baseline monsoon behaviour onto which Tibetan growth and other regional topographic changes (Iranian Plateau, East African highlands) act as amplifiers. As continents separate, enhanced zonal asymmetries and broader tropical ocean fetch increase the potential for strong boreal-summer moisture transport into East Asia. Conversely, restricted basins, seaways and alternate gateway states can suppress low-level moisture supply and weaken the seasonality signal, even under elevated terrain. There is also a great deal of debate still on the relative position of India through time, when initial contact was first made, as well as the relative positions and extents of Greater India and associated microplates [65]. Our comparison of Aptian–Maastrichtian–Ypresian–Chattian slices highlights that similar annual precipitation totals between reconstructions can mask very different seasonal structures and mechanisms of monsoon precipitation, often traceable to differences in land–sea distribution that alter moisture pathways and the position/strength of the subtropical highs. More work is needed to better quantify this effect.

### Palaeogeographic uncertainty

Divergence among reconstructions arises from (i) different assumptions in plate kinematic models and topographic assembly [65,87–91], (ii) differing palaeoaltimetric methodologies (e.g. isotopic, enthalpy and lapse rates) and lapse-rate assumptions (including environmental versus topographic lapse rates and wet-bulb versus isotopic lapse-rate choices [71,72]), (iii) often unquantifiable heterogeneous assumptions of erosion and sediment redistribution [92], (iv) variable use/transparency of underlying geological data used [90,93] and (v) differing age constraints. Where proxy information is sparse, interpolation choices, for example, imposing a high plateau versus dual mountain-and-valley system, will produce different orographic dynamics for ascent and moisture transport. In addition, some palaeogeographies ensure time-consistent morphologic evolution, whereas others only consider best-available ‘snapshots’; the former requires more assumptions on plate convergent rates, mantle movement, weathering, etc. while the latter can lead to inconsistencies through time as topographic/bathymetric histories do not need to be considered or conserved. Different methodological choices may explain why Getech and Robertsons palaeogeographies simulate an earlier re-establishment of seasonal precipitation over the EASM region by the mid-Eocene, while in Scotese this does not occur until the mid-Miocene. This difference in EASM-region response is primarily driven by differences in the height, extent and latitudinal locations of Tibetan topographic evolution between reconstructions.

Consequently, palaeogeographic uncertainties (local, regional and global) can lead to some reconstructions being monsoonal, while others may be non-monsoonal. Ideally, proxy-palaeoclimate model comparisons would be framed against ensembles of boundary conditions rather than a single favoured reconstruction. Unfortunately, cost and technical limitations make this difficult, especially for multiple time periods.

A systematic approach is required to constrain the local, regional and global palaeogeographic uncertainty on EASM evolution. Specifically,

1. **Reducing local uncertainty**. Improved estimations of Tibetan topographic evolution (Himalaya, Gangdese, Qiangtang, Tian Shan, Hengduan) through community-driven, openly documented, terrane-level topographic histories with updated palaeoaltimetry (including energy conservation approaches), thermochronology, sediment provenance and quantification of (thermal and isotopic) lapse-rate choices [71,72]. Where significant disagreement in the growth model for Tibet exists, then accompanying topographic scenarios where the underlying data agree for all scenarios to allow model sensitivity tests to be performed (as is currently done by various modelling groups).
2. **Reducing regional uncertainty**. Greater focus on regional topographies and coastlines is required (retreat of the Paratethys, growth of the Iranian Plateau and East Africa and the position of (Greater) India). Recently, there has been greater recognition that regional palaeogeography, in particular the suturing of Arabian–Eurasia Plateau and topographic rise of the Iranian Plateau is important in modifying climate dynamics in the EASM region [11,38,41,43]. Palaeo-altimetric and chronological analyses to better constrain the timing of elevation changes are essential to help constrain Miocene ‘super-monsoons’ [11,94].
3. **Reducing global uncertainty**. The impact of non-Asian changes in palaeogeography (land–sea distribution, topography, ice sheet growth) is highly unconstrained. This requires quantification of the robustness of distant mountain ranges such as the Andes, Alps, Rockies, Sierra Nevada, etc., specifically their spatial extent and elevation. Community-led projects such as (PlateauPlus, TopoEurope, TopoAsia) will aid in reducing global uncertainties. This will allow greater quantification of teleconnections in deep-time and their impact on the evolving EASM.

A further source of uncertainty concerns the implementation of a given palaeogeography within the palaeoclimate model, in particular model resolution and ability to represent monsoon dynamics. Although the relatively coarse resolution of the HadCM3 model is a clear limitation for resolving local orographic precipitation and coastal processes, its implications must be interpreted carefully. Coarse grids inevitably smooth topographic gradients and may weaken local precipitation contrasts; however, at broader regional scales, such as East Asia, the lack of finer scale topographic features is not a hinderance [95]. A critical improvement to models (N48 resolution (∼300 km grid box) or better) is the ability to better resolve precipitation dynamics, particularly for mid-latitude disturbances, but even such improvements still require parameterisations for convection and cloud microphysics. Convection-resolving simulations would require global kilometre-scale resolution, far beyond the practical scope of coupled deep-time palaeoclimate experiments, especially to achieve equilibrations across multiple time periods and sensitivity experiments. In this context, model fidelity in reproducing the mean state of the modern East Asian monsoon is a more relevant benchmark than nominal spatial resolution alone. Model performance against modern observations for the East Asian monsoon indicate how skilful a model is at representing the combined Earth system dynamics of the region (e.g. Sperber *et al.* [95]) as well as the influence of teleconnections that might influence a monsoon mean state. Consequently, while palaeogeographic resolution-related limitations should be acknowledged, depending on the spatial scale being investigated, the ability of the model to accurately reproduce the behaviours driving monsoon dynamics is more important.

Palaeogeographic forcing and its impact on the EASM could also be further modified through linear and non-linear external and internal forcings. For example, it is well known that orbital forcing can modulate the seasonal length, latitudinal distribution and intensity of the EASM. However, interactions between different continental configurations, sea levels, Asian/Tibetan topographic heights, bathymetries and terrestrial ice sheet heights and/or extents and orbital forcing could also dissimilarly impact the behaviour of the EASM between reconstructions leading to different sensitivities. Likewise, teleconnections from distant mountains can also impact on the EASM. As an example, a low elevation Rocky Mountains can lead to greater moisture transport from the Pacific into the Atlantic. This can freshen the North Atlantic leading to a reduction in the strength of even collapse in the Atlantic Meridional Ocean Circulation [96], which weakens the EASM [97]. Changes in bathymetry and ocean gateways can also lead to changes in large-scale ocean circulations which can also directly impact the response of the EASM, this could also be further compounded by different palaeogeographies having different orbital forcing and orographic sensitivities. Together, this highlights the importance of constraining palaeogeographic forcing through testing different realisations to better understand their impact on the EASM system.

## CONCLUSIONS

Our multi-reconstruction simulations demonstrate that both the evolution and intensity of monsoon dynamics in the modern EASM region are highly contingent on palaeogeography. Although the Getech palaeogeography appears to better reproduce the annual hydrological signal as inferred from proxy data, this agreement likely arises from its assumption of broader and higher Tibetan elevations during the Cenozoic rather than from a demonstrably more accurate reconstruction of Tibetan orographic evolution. Assessment is further hindered by the paucity of seasonally resolved quantitative proxy records with adequate temporal and spatial coverage for deep-time intervals. Most geological proxies are inadequate for capturing critical monsoon indicators such as seasonal precipitation measures and when that precipitation occurs within the annual cycle. This limitation is particularly important given how rapidly our understanding of Tibetan topographic evolution has changed over the past 5–10 years, including evidence for a broad dual-mountain system flanking a relatively low central valley through much of the Palaeogene [59–61], a configuration not represented in many earlier palaeogeographies. Disagreement among reconstructions arises from multiple, interacting sources (plate kinematic models and terrane assembly; palaeoaltimetric datasets and lapse-rate choices; heterogeneous assumptions about erosion, sediment redistribution; data opacity and density of underlying geological constraints; and differing time-sampling strategies). These all lead to different EASM evolution histories.

Previous research has predominantly focused on local Tibetan evolution; however, there is growing realisation that regional (PTH) and global changes in palaeogeography can also have a first-order impact on monsoon behaviour in the EASM region [11,41,83].

Tantalisingly, the suites of simulations investigated here potentially demonstrate that land–sea configuration provides the long-term baseline, given the general similarity between all three palaeogeographies, upon which local and regional mountain building acts to amplify (as has been shown previously with the Tibetan Plateau [7,11,12,38,58,59,63,85,87,98]) monsoon behaviour in the EASM region. However, it is not so simple because continental breakup increases zonal asymmetry and tropical ocean fetch, preconditioning the system for stronger monsoon season moisture transport into the EASM region, as shown by the aridification in the mid-to-Late Cretaceous. The direct impact of land–sea distribution over geologic timescales on monsoons the EASM region still remains to be tested. During the Cenozoic, the timing of Tibetan mountain building ultimately dictates when monsoon conditions push further into the continent, as well as their intensity. There is also now greater awareness that changes in global palaeogeography in distant regions can have a teleconnected impact on the climate of the EASM region that may either enhance or disrupt monsoon dynamics. A consistent feature in all three mid-to-Late Cretaceous reconstructions is that they are all arid, yet they have very different local topographies.

Improved constraints on local, regional and global palaeogeography, in particular topography, through community-led open-source databases will help constrain uncertainties improving our understanding of EASM evolution and its drivers and mechanisms (e.g. thermal versus mechanical forcing).

## MATERIALS AND METHODS

### Palaeoclimate model

We used the fully coupled AOGCM HadCM3BL-M2.1aD [100] at 2.5° × 3.75° (latitude × longitude) with 19 atmospheric and 20 ocean levels. The ocean is a 3-D primitive-equation model with centred advection and a second-order scheme. Standard flux adjustments are applied to avoid long-term drift in multi-millennial integrations. Sea ice uses a zero-layer thermodynamic scheme with fractional grid-cell coverage. Land surface processes are represented by MOSES 2.1 [101] with a globally uniform medium-loam soil. Dynamic vegetation is simulated by top-down representation of interactive foliage and flora including dynamics, which prognoses fractional cover of five plant functional types (C3 grass, C4 grass, shrub, broadleaf tree, needleleaf tree).

Cloud microphysics follow Sagoo *et al.* [102] and Kiehl and Shields [103], adjusting cloud condensation nuclei and droplet effective radius to better represent high-latitude warmth while retaining realistic PI climatology. Simulations were initialised from equilibrated PI states and run to equilibrium (>5000 model years; often >10 000). Equilibrium criteria required: (i) global, volume-mean ocean temperature drift < 1°C per 1000 years, (ii) surface-air temperature trend < 0.3°C per 1000 years and (iii) top-of-atmosphere net energy imbalance < 0.25 W/m^2^ averaged over the final 100 years. For the Scotese series, the climate model configuration is identical to that in Judd *et al.* [104] and Malanoski *et al.* [105].

### Boundary conditions and experiments

To assess palaeogeographic controls (topography, bathymetry and land–sea distribution) on the EASM, we conducted time-slice simulations from 145 to 0 Ma using three Digital Elevation Model (DEM) suites: Getech Plc, Scotese (PALEOMAP) and Robertson Plc. Getech provides stage-based, time-consistent reconstructions (∼30 slices); Scotese provides time-consistent 5 Myr slices (∼29); Robertson provides stand-alone snapshots (∼10). Each DEM includes land–sea masks, orography/bathymetry and prescribed ice sheets. Native grids are 0.5° (Getech), 1° (Scotese) and 0.1° (Robertson); fields were bilinearly remapped to the model grid (3.75° × 2.5°) with derived sub-grid orographic parameters. While all products incorporate geological constraints, underlying data and priors are often not uniformly documented.

Atmospheric *p*CO₂ for Getech and Scotese followed multi-proxy reconstructions of Foster *et al.* [99] for 145–69 Ma and Rae *et al.* [82] for 69–0 Ma. Robertsons used more idealised *p*CO₂ that fall within uncertainty ranges of Foster *et al.* [99] for 145–69 Ma and Rae *et al.* [82]; see Fig. 1c for details. While this is inconsistent with the Getech/Scotese simulations, it provides valuable insight with the addition of another palaeogeography and as highlighted in Farnsworth *et al.* [106] *p*CO_2_ over the geologic record is only a second-order impact on the EASM. The PI control used 280 ppm. Time-dependent solar luminosity was prescribed from Gough [107] (PI reference 1361 W/m^2^).

### Palaeomonsoon proxy data

Model–data evaluation used the unchanged compilation in Farnsworth *et al.*, combining plant/animal assemblages and other climatically sensitive terrestrial and marine indicators interpreted for precipitation. Details and citations are provided in the Supplementary Information of Farnsworth *et al.* [11].

Detailed methods and materials can be found in Supplementary Information.

## Supplementary Material

nwag534_Supplemental_File

## Data Availability

All data needed to evaluate the conclusions in the paper are present in the paper and/or Supplementary data. Model data can be accessed from the repository at www.bridge.bris.ac.uk/resources/simulations.
