# Supplementary material for: What was the role of palaeogeography in East Asian monsoon evolution since the Cretaceous?
Source: Natl Sci Rev. 2026 Sep 4;13(17):nwag534. doi: 10.1093/nsr/nwag534 (PMC13613981; doi:10.1093/nsr/nwag534)
Supplement: Supplementary file 1 [file nwag534_supplemental_file.pdf]

Supplementary information: Palaeogeographic uncertainty reveals divergent trajectories of the East Asian monsoon over 145 Myr

Authors: Farnsworth A[1,2,*]., Lunt D.J.[1], Valdes P.J[1]., Spicer R.A[3]., Robinson S.A[4]., Ding L.[2], Xiong X.[2], Witkowski C.[5,6], Scotese C.[7], Markwick P.[8], Pancost R[5,6]

[1]School of Geographical Sciences and Cabot Institute for the Environment, University of Bristol, Bristol, UK

[2]State Key Laboratory of Tibetan Plateau Earth System, Environment and Resources (TPESER), Institute of Tibetan Plateau Research, Chinese Academy of Sciences, Beijing, China

[3]School of Environment, Earth and Ecosystem Sciences, The Open University, Milton Keynes MK7 6AA, UK.

[4]School of Earth Sciences, and Cabot Institute, University of Bristol, Bristol, BS8 1TS, UK

[5]Organic Geochemistry Unit, School of Chemistry, University of Bristol, Bristol, BS8 1QU, UK

[6]Department of Earth and Planetary Sciences, Northwestern University, Evanston, IL 60208, USA

[7]Knowing Earth Limited, Otley, West Yorkshire, UK

Contents:

1. Material and methods
2. Supplementary Figures S1-S16

1. Materials and Methods

a. Palaeoclimate model.

We utilized a fully coupled atmosphere-ocean general circulation model (HadCM3BL-M2.1aD)[1] with a spatial resolution of 2.5 × 3.75° latitude by longitude incorporating 19 vertical levels in the atmosphere and 20 vertical levels in the ocean. HadCM3BL-M2.1aD has shown skill in reproducing both modern, Quaternary and deep-time climates compared to observations (see Valdes et al., 2017 for details[1]). A predictive interactive vegetation scheme using modern expressions for broadleaf trees, deciduous trees, shrubs, and grasses and a globally uniform distribution of a medium loam soil characteristics are used in the model land surface scheme (MOSES 2.1)[2]. This is supplemented with the interactive vegetation scheme, TRIFFID (Top-Down Representation of Interactive Foliage and Flora Including Dynamics), which predicts global vegetation cover using five (C3, grass, C4 grass, Shrub, Broadleaf tree, Needleleaf tree) Plant Functional Types (PFTs). The ocean model is based on the model of Cox et al. (1984)[3] and is a full primitive equation, 3D model of the ocean. A second-order numerical

scheme is used along with centered advection to remove nonlinear instabilities. Flux adjustments (such as artificial heat and salinity adjustments in the ocean component model[4] are applied to prevent drifting to unrealistic values, which is crucial for long paleoclimate simulations. Sea ice is calculated on a zero-layer model with partial coverage, and a consistent salinity is assumed for ice. Each simulation was initialized from an equilibrated pre-industrial state in the atmosphere and ocean and run until full system equilibrium has been established and run for over >5,000 model years for each series (often >10,000). Equilibrium here is defined as: i) The globally and volume-integrated annual mean ocean temperature trend is less than 1°C per 1000 years; ii) trends in surface air temperature are less than 0.3°C per 1000 years; iii) net energy balance at the top of the atmosphere, averaged over a 100-year period at the end of the simulation, is less than 0.25/W m2.

The model includes a recent update which modifies cloud condensation nuclei density and cloud droplet effective radius to better represent high-latitude warmth as highlighted by proxy-observations using the methods of Sagoo et al.(2013)[5] and Kiehl and Shields (2013)[6] allowing a more representative pole-to-Equator temperature gradient to develop under warmer climates of the past, while still producing a skillful Pre-industrial climate.

HadCM3 shows good skill in simulating the mean climate state in good agreement with observations in both surface temperature, precipitation, 850mb winds and mean sea level pressure seasonally and annually in both spatial and temporal distributions in East Asia[7-9].

It was found by Jiang et al. (2005)[9] that HadCM3 performs better in the East Asian region relative to many other contemporary coupled climate models, and even performs better than the multi-model mean. When the regional average precipitation in East Asia was compared against modern in-situ observations (CRU observations) for June-August, HadCM3 produced values of 3.95 mm/day^{-1} compared with 3.94 mm/day^{-1} in the observations[9]. However, there is a small positive bias in the HadCM3L (pre-industrial simulation) model in annual precipitation compared to CMAP observations in the monsoon region (4.3 mm/day^{-1} and 3.7 mm/day^{-1}, respectively). Comparison of the pre-industrial simulation against 1901–1930 Global Precipitation Climatology Centre (GPCC) rain gauge analysis product[10], confirms this positive bias (~0.3-0.5mm/day^{-1}) on an annualised and seasonal (Jun-Aug) basis (not shown).

I. Model boundary conditions and sensitivity studies

Digital Elevation Models. To ascertain the impact of Palaeogeographic forcing on the East Asian Summer Monsoon (EASM) a series of past climate simulations using three different Digital Elevation Models (DEMs) were run for Getech Plc (Fig. S1)., Scotese (Fig. S2), and Robertsons Plc. (Fig. S3) from the beginning of the Cretaceous (145 Ma) to the Modern (0 Ma). Getech Plc have representative time periods for each geologic stage (30 unique time periods). Scotese (PALEOMAP[11]) reconstructs time periods every 5 million years (29 unique time periods). Robertsons Plc have fewer representative time periods (10 unique time periods). Each DEM comprises a land sea mask, topographic and bathymetric information as well as ice sheet extent. Each reconstruction utilises the GPlates system to compute the convergent and divergent histories of each tectonic plate. Both Getech Plc and Scotese DEMs are time consistent in that adjacent time periods incorporate information from the previous time period to ensure a consistent evolution of topography and bathymetry towards the Modern. Robertsons on the other hand are stand-alone reconstructions where adjacent time periods topographic and bathymetric information are not used to inform each time period. While, all reconstructions incorporate a series of geologic proxy-data in the development to evaluate and aid each reconstruction, the underlaying data for each is unfortunately not published or documented.

Each palaeogeographic series DEM has a native resolution of 0.5°x0.5° for Getech, 1°x1° for Scotese and 0.1°x0.1° for Robertsons. From these we generated model-resolution (3.75°x2.5°) land-sea mask, topography and bathymetry, and the sub-gridscale orographic variables required by the model.

Atmospheric carbon dioxide concentrations for both Getech Plc and Scotese, realistic proxy-CO_2 is derived from Rae, et al. (2021)[12] between 69 Ma – 0 Ma. For the Cretaceous simulations between 145 Ma to 69 Ma, proxy-CO_2 reconstructions from Foster et al. (2017)[13] are utilised. Robertsons has more idealised pCO_2 also within range of uncertainty of proxy-CO2 estimates which should be noted will produces a slight inconsistency when comparing against both Getech and Plc and Scotese. However, with CO_2 forcing a secondary factor in determining the behaviour of the EASM this will likely not pose a critical impact on the results. The Pre-industrial simulation has CO_2 set at 280 ppm.

Solar forcing. Each simulation has time dependant solar luminosity (W/m^2) as calculated by Gough (1981)[14] from a 1361 W/m2 starting point for the Pre-industrial. As a rough estimate, solar luminosity is increase at a rate of +1% W/m2 every 100 million years.

Pan-Tibetan Highlands are unmodified in each palaeogeographic reconstruction and represent a set of assumptions made by each plate modelling group in terms of how the Indian plate and other plate tectonics regionally have interacted over the Asian region since the beginning of the Cretaceous.

II. Palaeomonsoon proxy data.

The proxy-data (Fig. 1a & 2a) used is a combination of studies from plant flora and fauna and other indirect climatically sensitive terrestrial and marine proxies used to derive precipitation estimates. This compilation is unchanged and the same as in Farnsworth et al. (2019)[8], for more information please refer to Supplementary information in Farnsworth et al. (2019)[8].

III. Diagnostics

We analysed seasonal precipitation, the Monsoon Domain (MD) and Monsoon Precipitation Index (MPI) to diagnose monsoon structure and mechanisms for the paleo-rotated modern EASM domain from Getech for each palaeogeography. This was chosen because Robertsons Plc. Does not provided paleorotations making this more consistent across all three palaeogeographies. In particular, both Getech and Robertsons show no latitudinal variation between one another in its latitudinal location. Some manual latitudinal adjustment was undertaken for the rotations application to the Scotese palaeogeography due greater latitudinal movement of the Asian containment in the early-to-mid-Cretaceous.

Supplementary Figures:

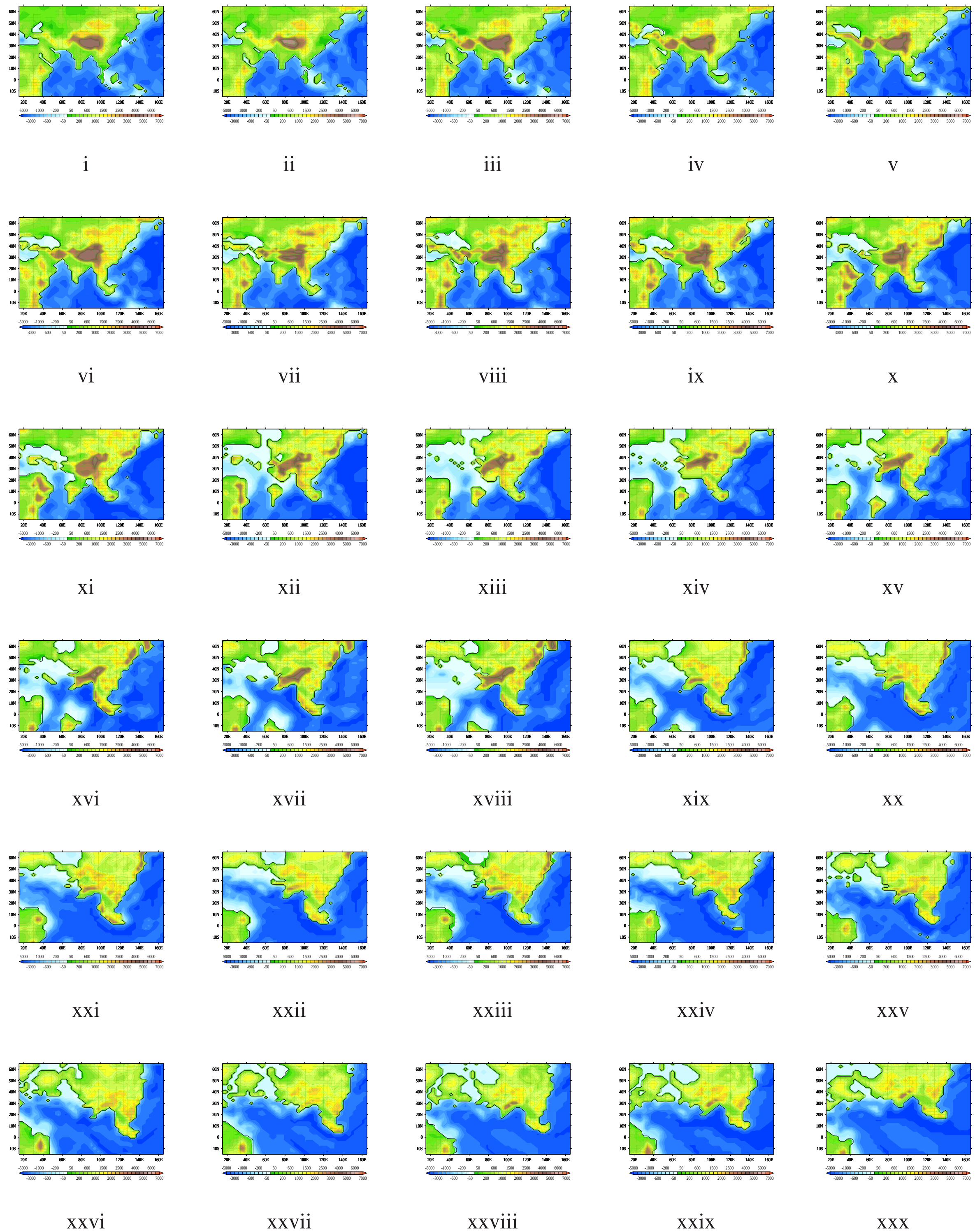

Figure S1 | **Getech Palaeogeography** in (i) Gelasian, (ii) Piacenzian, (iii) Zanclean, (iv) Messinian, (v) Tortonian, (vi) Serravallian, (vii) Langhian, (viii) Burdigalian, (ix) Aquitanian, (x) Chattian, (xi) Rupelian, (xii) Pribonian, (xiii) Bartonian, (xiv) Lutetian, (xv) Ypresian, (xvi) Thanetian, (xvii) Selandian, (xviii) Danian, (xix) Maastrichtian, (xx) Campanian, (xxi) Santonian, (xxii) Coniacian, (xxiii) Turonian, (xxiv) Cenomanian, (xxv) Albian, (xxvi) Aptian, (xxvii) Barremian, (xxviii) Hauterivian, (xxix) Valanginian, (xxx) Berriasian simulations.

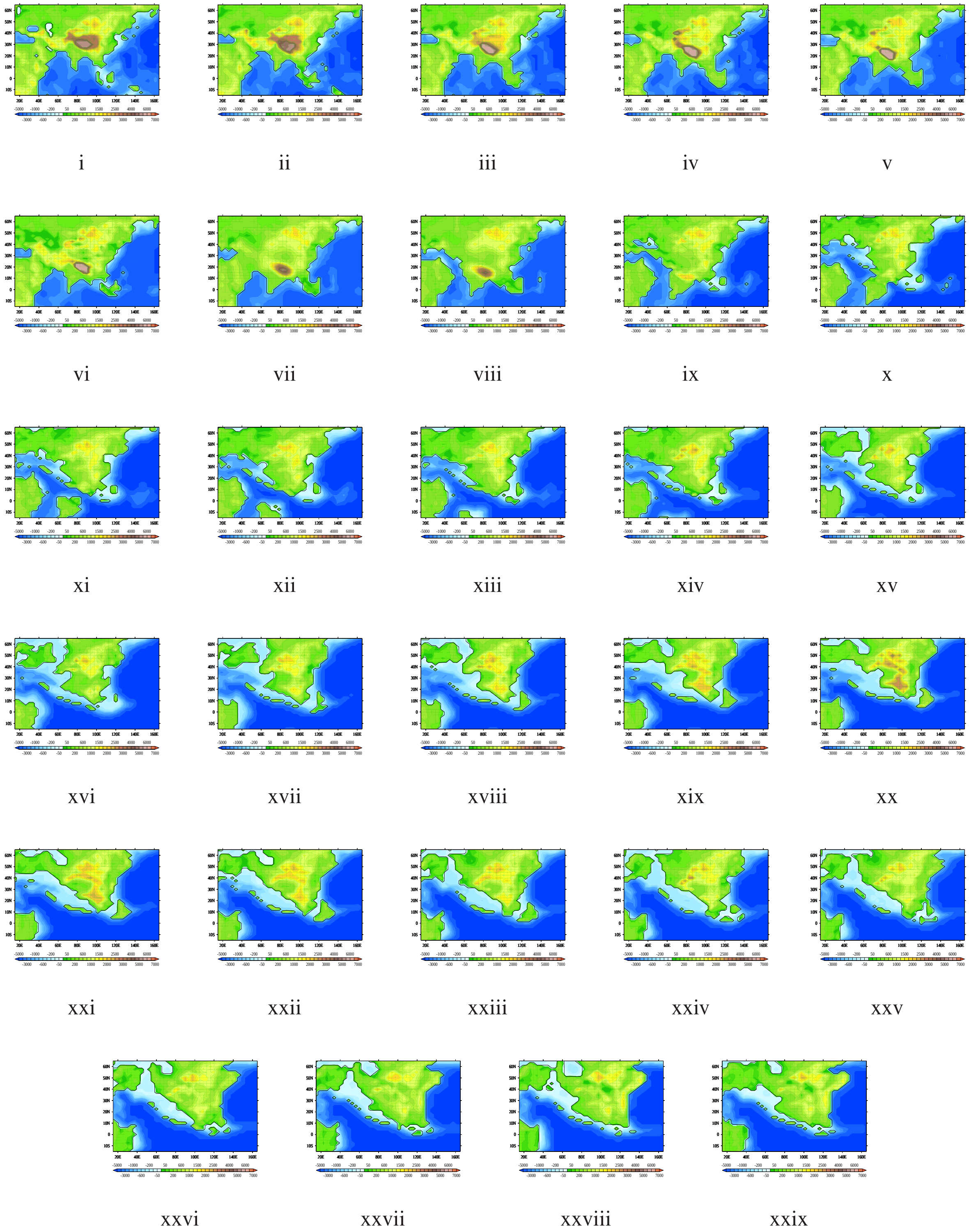

Figure S2 | Scotese Palaeogeography in (i) Zanclean, (ii) Tortonian, (iii) Langhian, (iv) Burdigalian, (v) Chattian, (vi) Rupelian, (vii) Priabonian, (viii) Bartonian, (ix) Lutetian, (x) Ypresian, (xi) Thanetian, (xii) Selandian, (xiii) Danian, (xiv) Maastrictian, (xv) Late Campanian, (xvi) Early Campanian, (xvii) Santonian, (xviii) Coniacian, (xix) Turonian, (xx) Cenomanian, (xxi) Late Albian, (xxii) Middle Albian, (xxiii) Early Albian, (xxiv) Late Aptian, (xxv) Early Aptian, (xxvi) Hauterivian, (xxvii) Valanginian, (xxviii) Barremian, (xxix) Berriasian simulations.

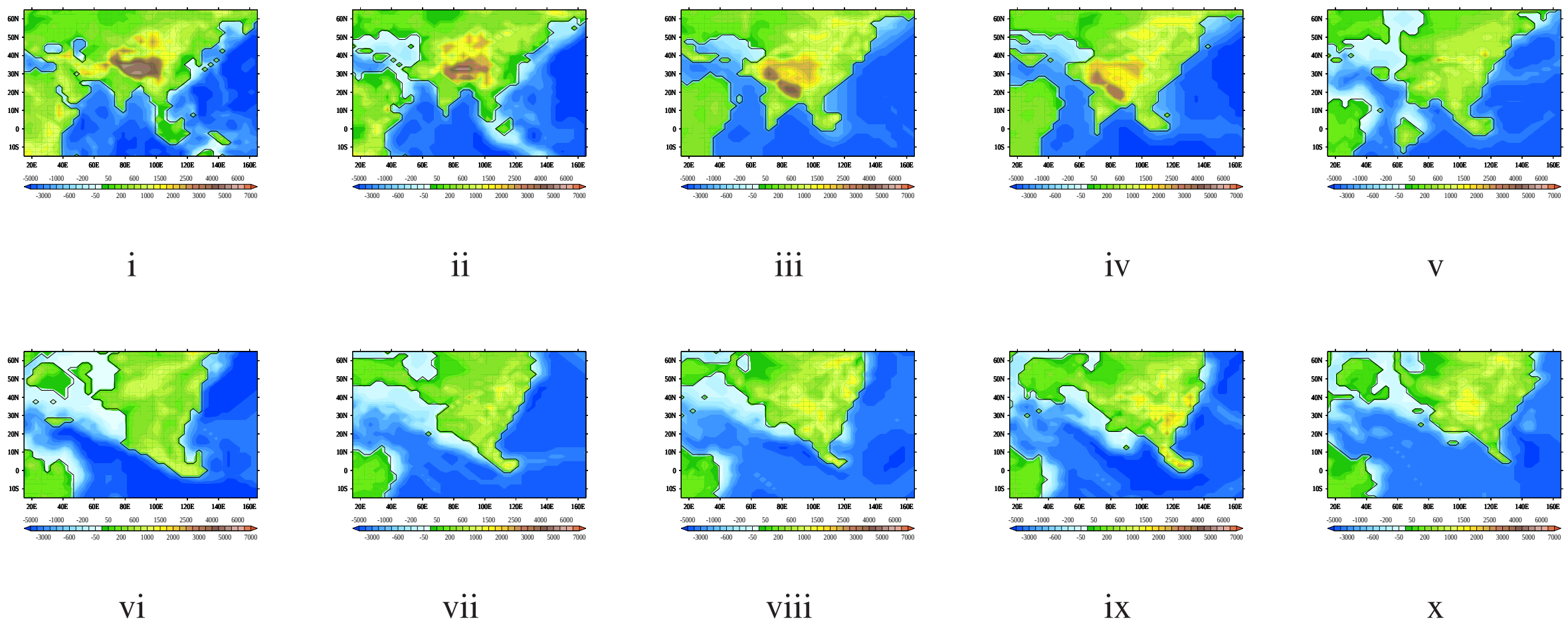

Figure S3 | Robertsons Palaeogeography in (i) Piacenzian, (ii) Serravallian, (iii) Chattian, (iv) Rupelian, (v) Ypresian, (vi) Maastrichtian, (vii) Turonian, (viii) Albian, (ix) Aptian, (x) Berriasian simulations.

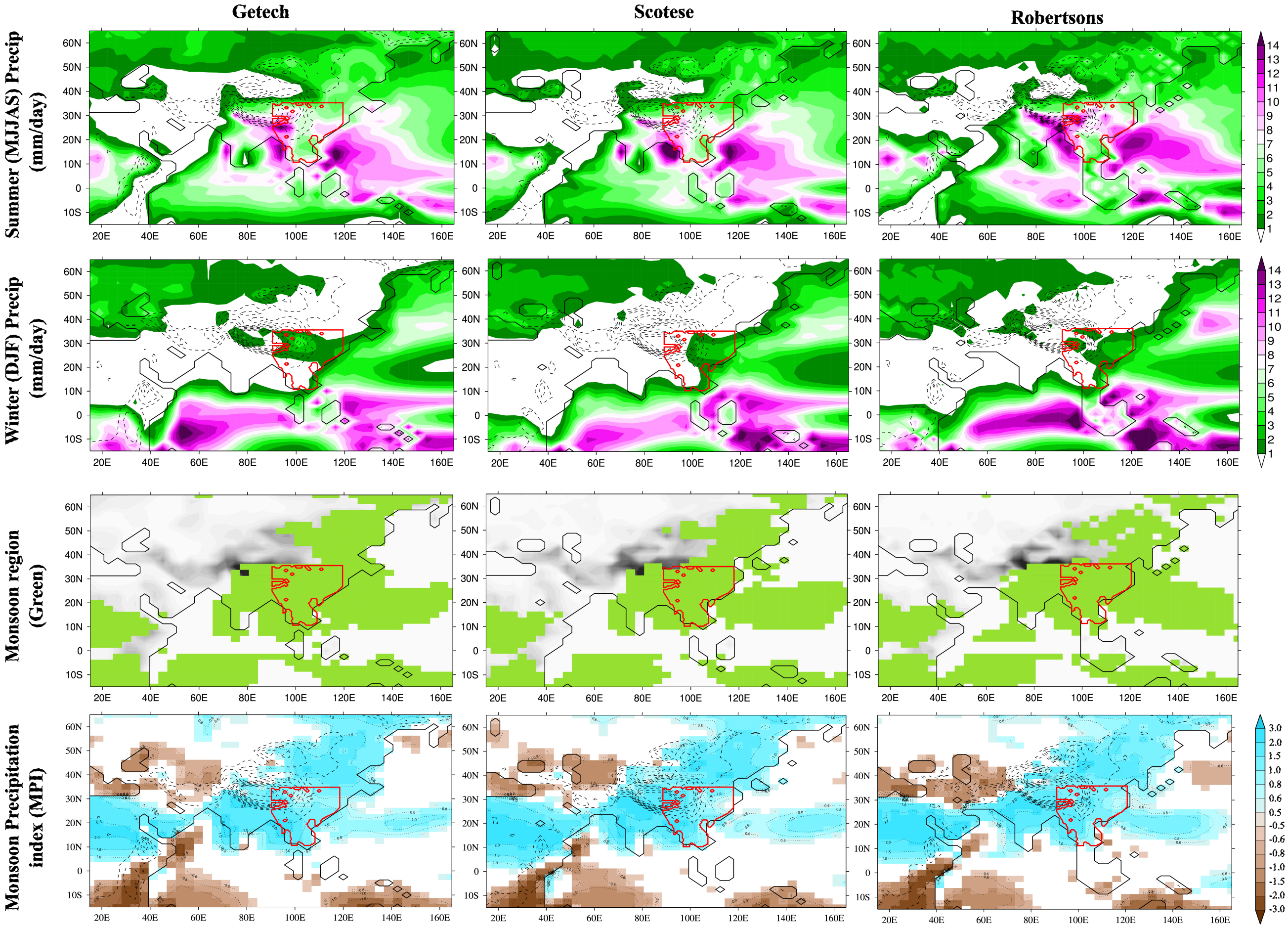

Figure S4. Pliocene (2.5-5.3 Ma) monsoon statistics. Summer May-September (MJJAS) precipitation (mm/day), Winter December-February (DJF), Monsoon region (green area) and Monsoon Precipitation Index (MPI) for i) Getech, ii) Scotese and Robertsons palaeogeographic series. Red region designates the modern rotated East Asian monsoon region.

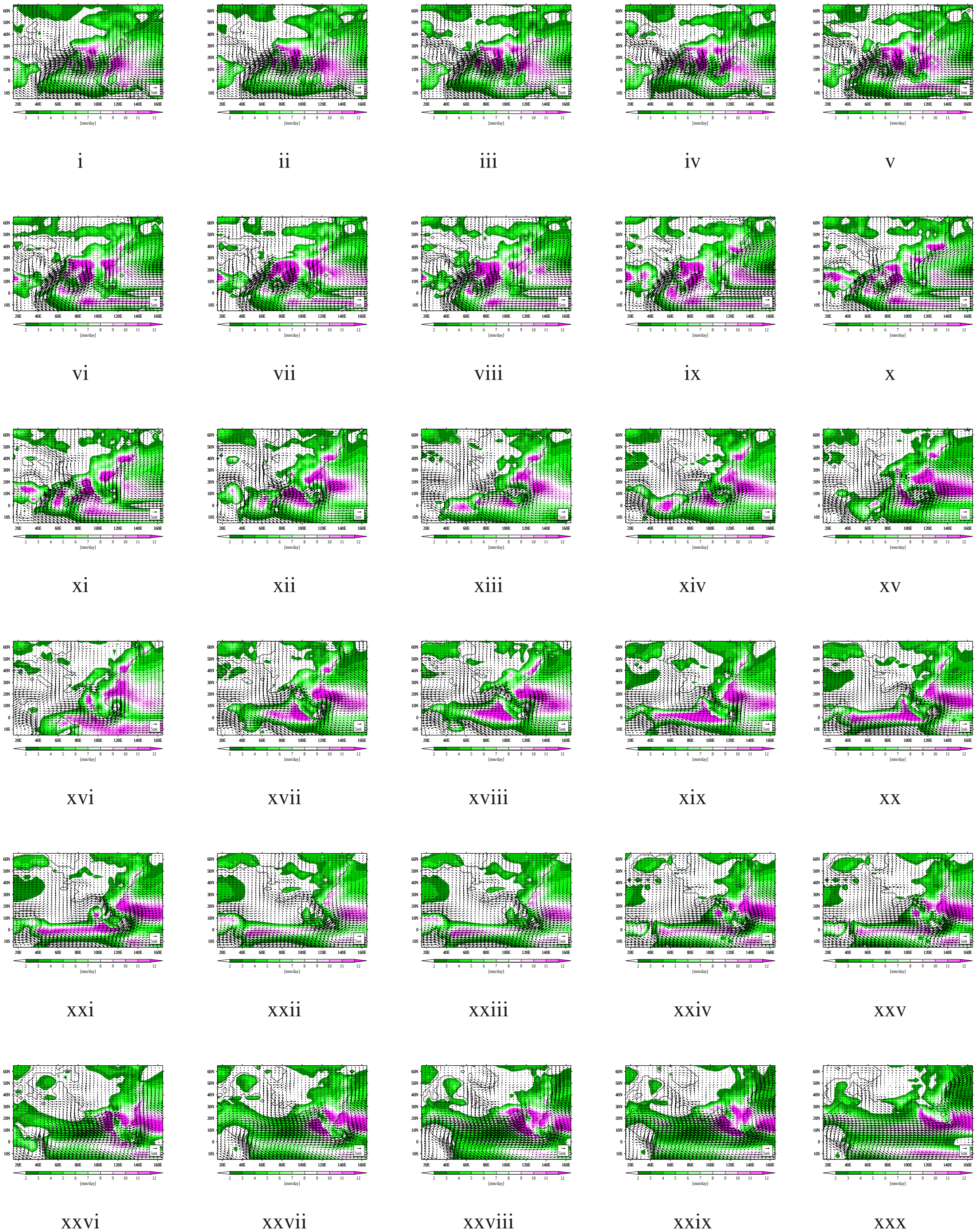

Figure S5 | Getech Palaeogeography JJA precipitation (mm/day) and 850 mb wind (m/s) in (i) Zanclean, (ii) Tortonian, (iii) Langhian, (iv) Burdigalian, (v) Chattian, (vi) Rupelian, (vii) Priabonian, (viii) Bartonian, (ix) Lutetian, (x) Ypresian, (xi) Thanetian, (xii) Selandian, (xiii) Danian, (xiv) Maastrictian, (xv) Late Campanian, (xvi) Early Campanian, (xvii) Santonian, (xviii) Turonian, (xix) Cenomanian, (xx) late Albian, (xxi) middle Albian, (xxii) early Albian, (xxiii) late Aptian, (xxiv) early Aptian, (xxv) Barremian, (xxvi) Hauterivian, (xxvii) Valanginian, (xxviii) late Barremian, (xxix) early Berriasian simulations.

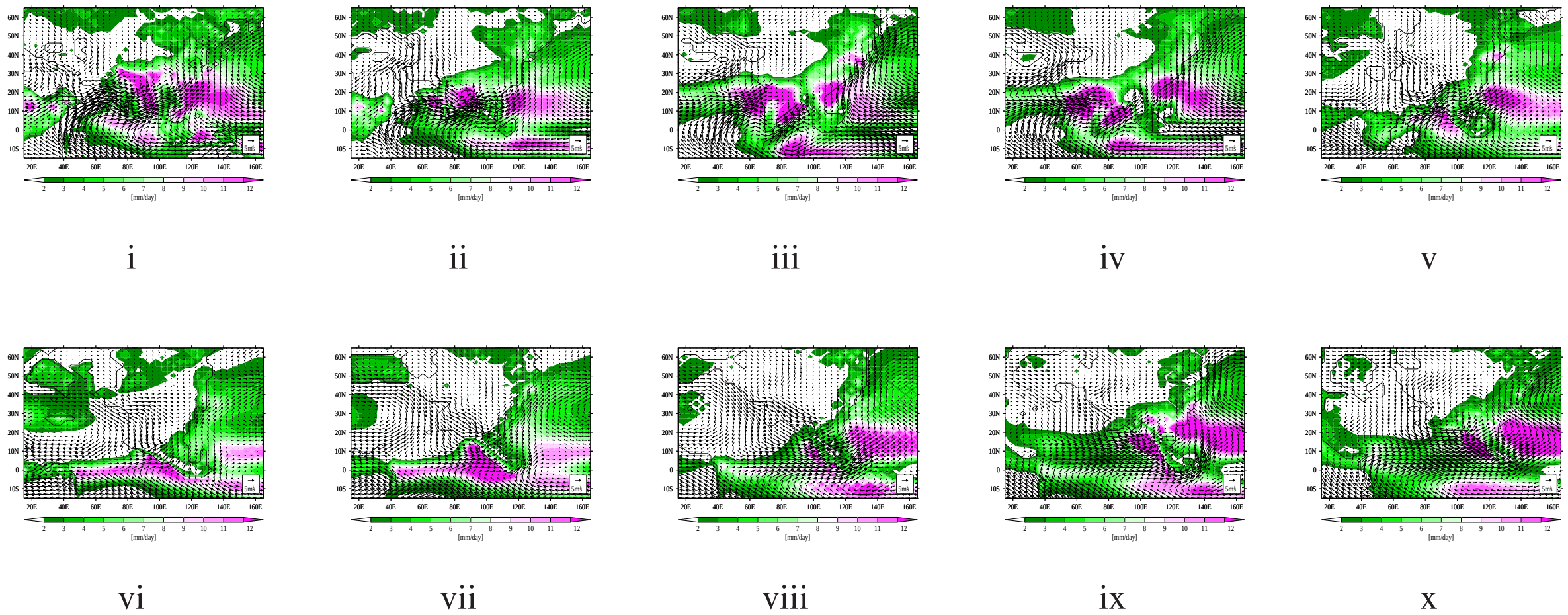

Figure S6 | Robertsons Palaeogeography JJA Precipitation (mm/day) and and 850 mb wind (m/s) in (i) Piacenzian, (ii) Serravallian, (iii) Chattian, (iv) Rupelian, (v) Ypresian, (vi) Maastrichtian, (vii) Turonian, (viii) Albian, (ix) Aptian, (x) Berriasian simulations.

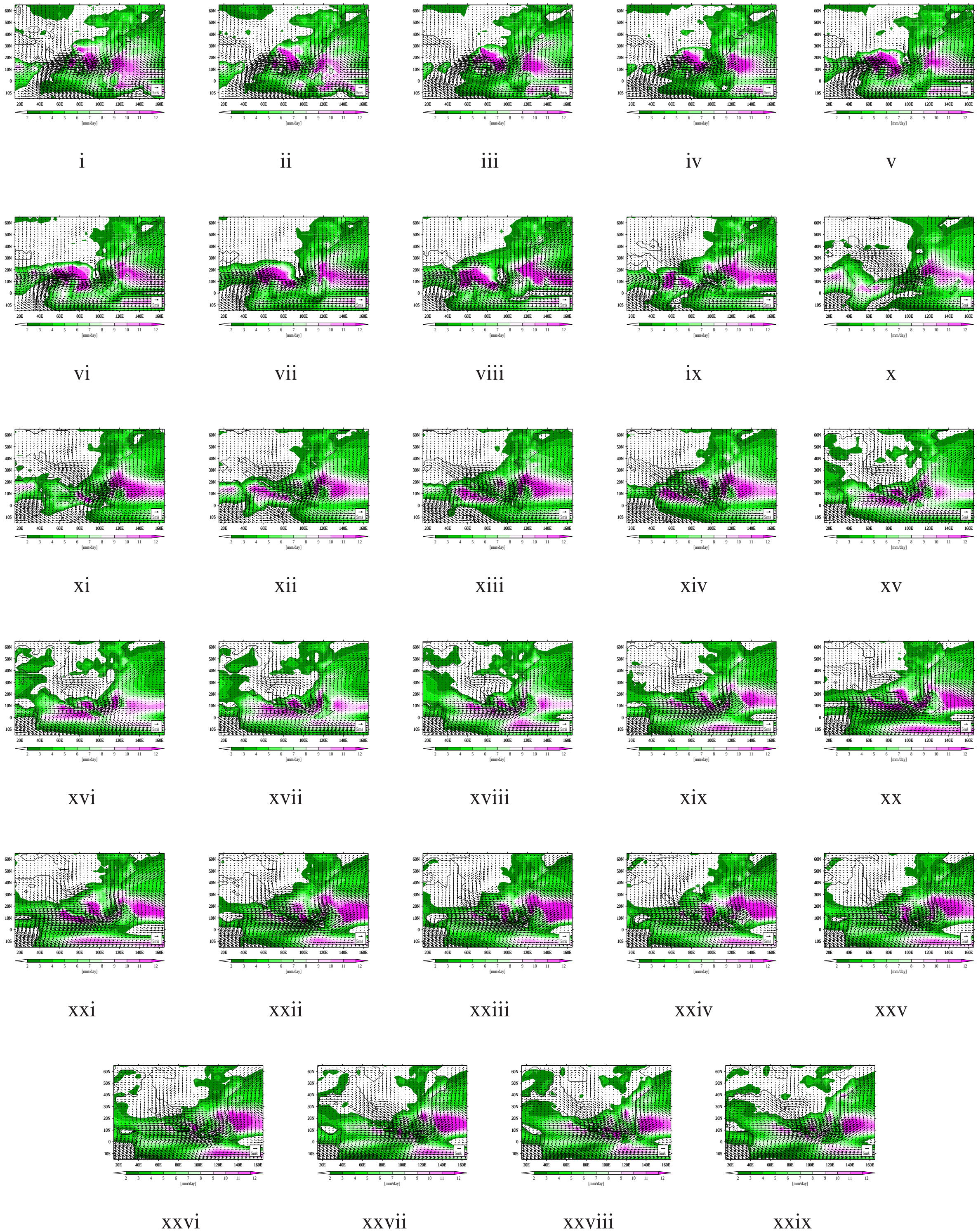

Figure S7 | Scotese Palaeogeography JJA Precipitation (mm/day) and and 850 mb wind (m/s) in (i) Zanclean, (ii) Tortonian, (iii) Langhian, (iv) Burdigalian, (v) Chattian, (vi) Rupelian, (vii) Priabonian, (viii) Bartonian, (ix) Lutetian, (x) Ypresian, (xi) Thanetian, (xii) Selandian, (xiii) Danian, (xiv) Maastrictian, (xv) Late Campanian, (xvi) Early Campanian, (xvii) Santonian, (xviii) Turonian, (xix) Cenomanian, (xx) late Albian, (xxi) middle Albian, (xxii) early Albian, (xxiii) late Albian, (xxiv) early Aptian, (xxv) Barremian, (xxvi) Hauterivian, (xxvii) Valanginian, (xxviii) late Barremian, (xxix) early Berriasian simulations.

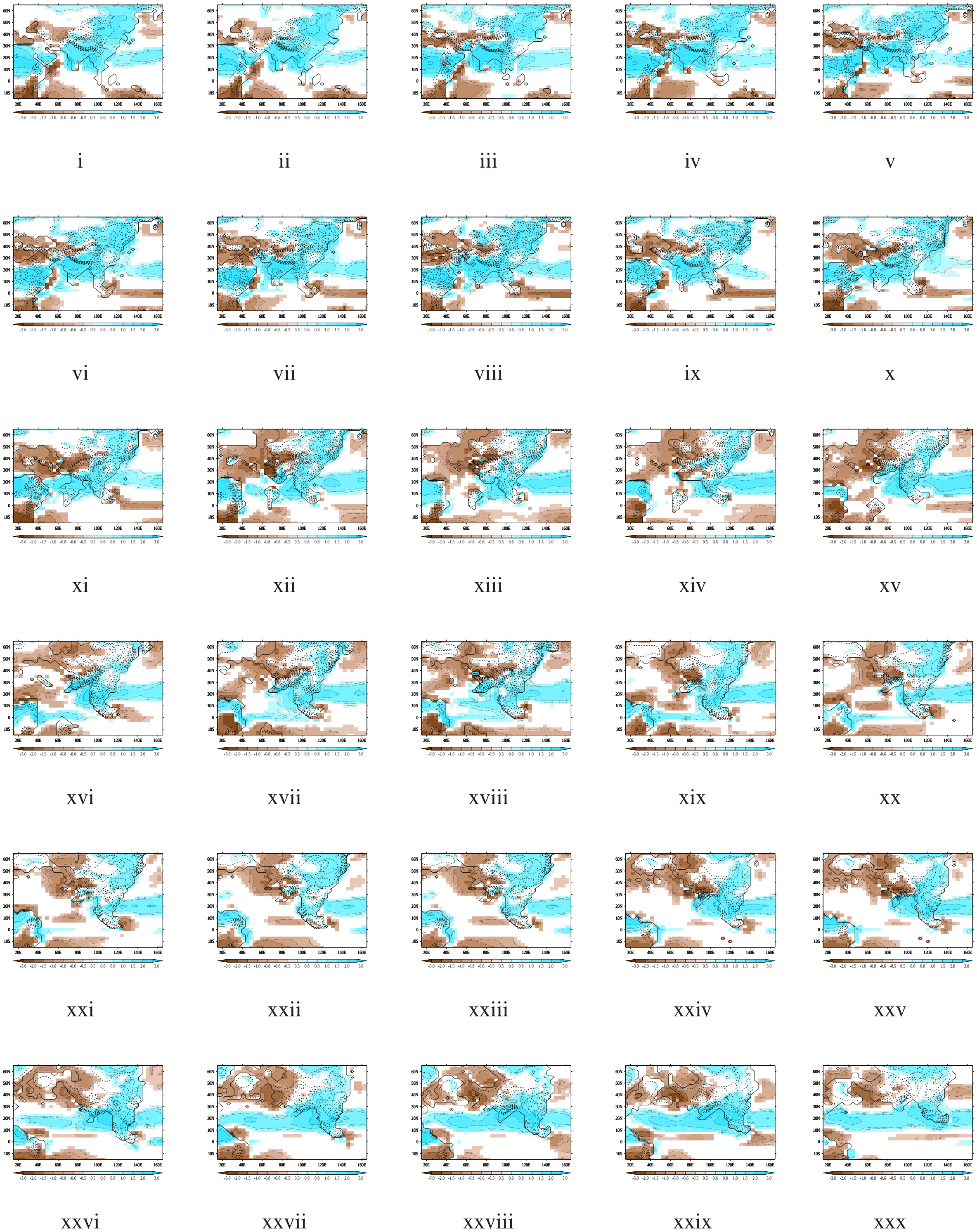

Figure S8 | Getech Palaeogeography Monsoon Precipitation Index in (i) Gelasian, (ii) Piacenzian, (iii) Zanclean, (iv) Messinian, (v) Tortonian, (vi) Serravallian, (vii) Langhian, (viii) Burdigalian, (ix) Aquitanian, (x) Chattian, (xi) Rupelian, (xii) Pribonian, (xiii) Bartonian, (xiv) Lutetian, (xv) Ypresian, (xvi) Thanetian, (xvii) Selandian, (xviii) Danian, (xix) Maastrichtian, (xx) Campanian, (xxi) Santonian, (xxii) Coniacian, (xxiii) Turonian, (xxiv) Cenomanian, (xxv) Albian, (xxvi) Aptian, (xxvii) Barremian, (xxviii) Hauterivian, (xxix) Valanginian, (xxx) Berriasian simulations.

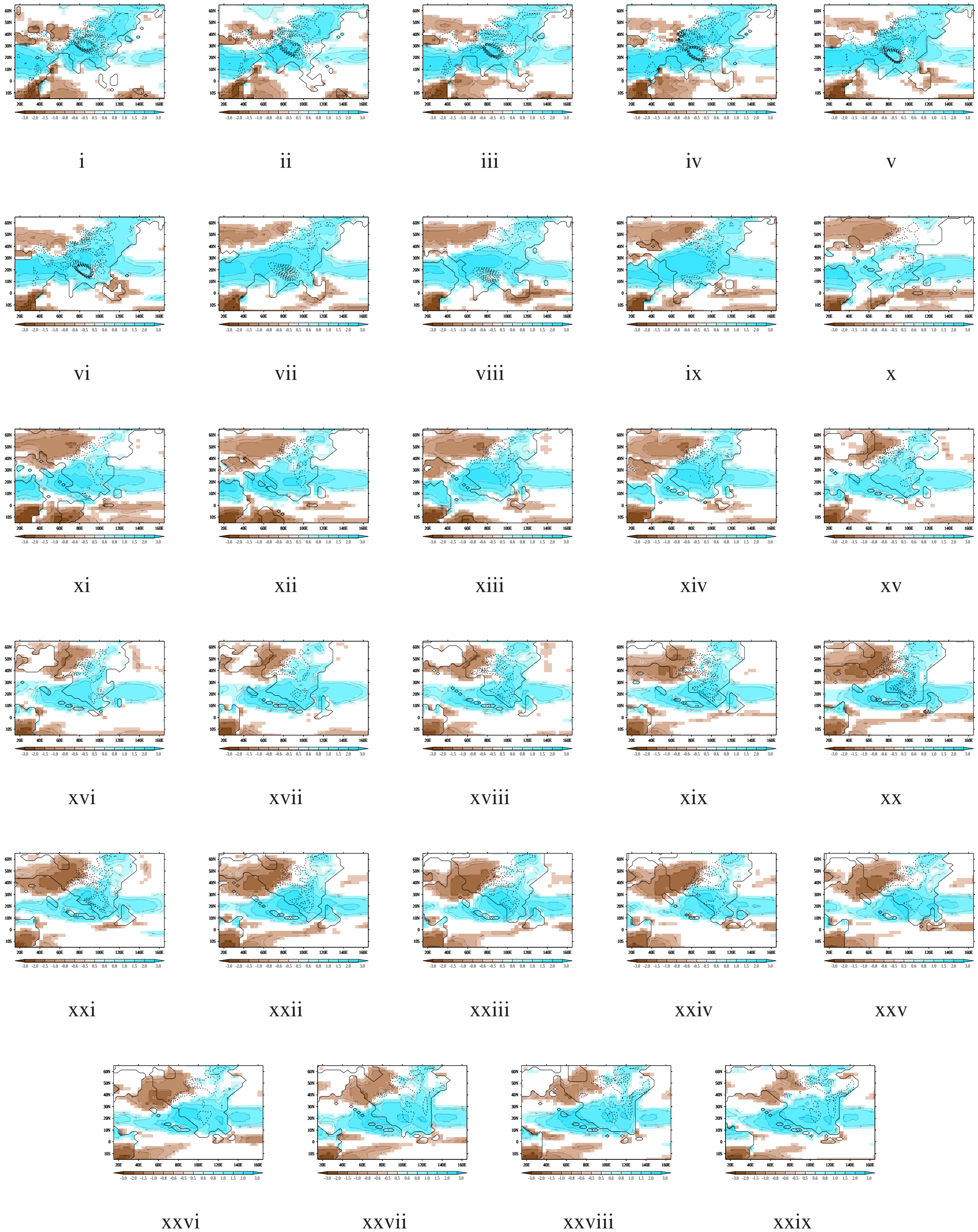

Figure S9 | Scotese Palaeogeography Monsoon Precipitation Index in (i) Zanclean, (ii) Tortonian, (iii) Langhian, (iv) Burdigalian, (v) Chattian, (vi) Rupelian, (vii) Priabonian, (viii) Bartonian, (ix) Lutetian, (x) Ypresian, (xi) Thanetian, (xii) Selandian, (xiii) Danian, (xiv) Maastrictian, (xv) Late Campanian, (xvi) Early Campanian, (xvii) Santonian, (xviii) Turonian, (xix) Cenomanian, (xx) late Albian, (xxi) middle Albian, (xxii) early Albian, (xxiii) late Aptian, (xxiv) early Aptian, (xxv) Barremian, (xxvi) Hauterivian, (xxvii) Valanginian, (xxviii) late Barremian, (xxix) early Berriasian simulations.

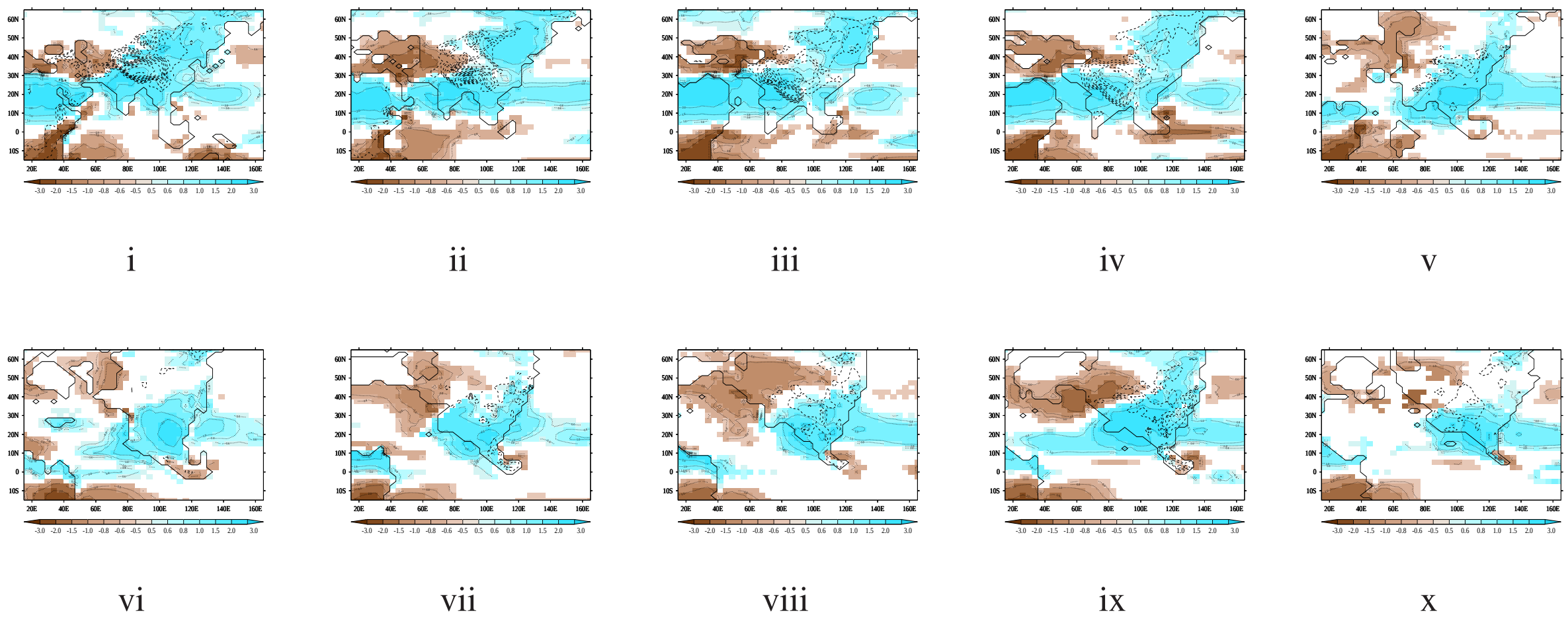

Figure S10 | Robertsons Palaeogeography Monsoon Precipitation Index in (i) Piacenzian, (ii) Serravallian, (iii) Chattian, (iv) Rupelian, (v) Ypresian, (vi) Maastrichtian, (vii) Turonian, (viii) Albian, (ix) Aptian, (x) Berriasian simulations.

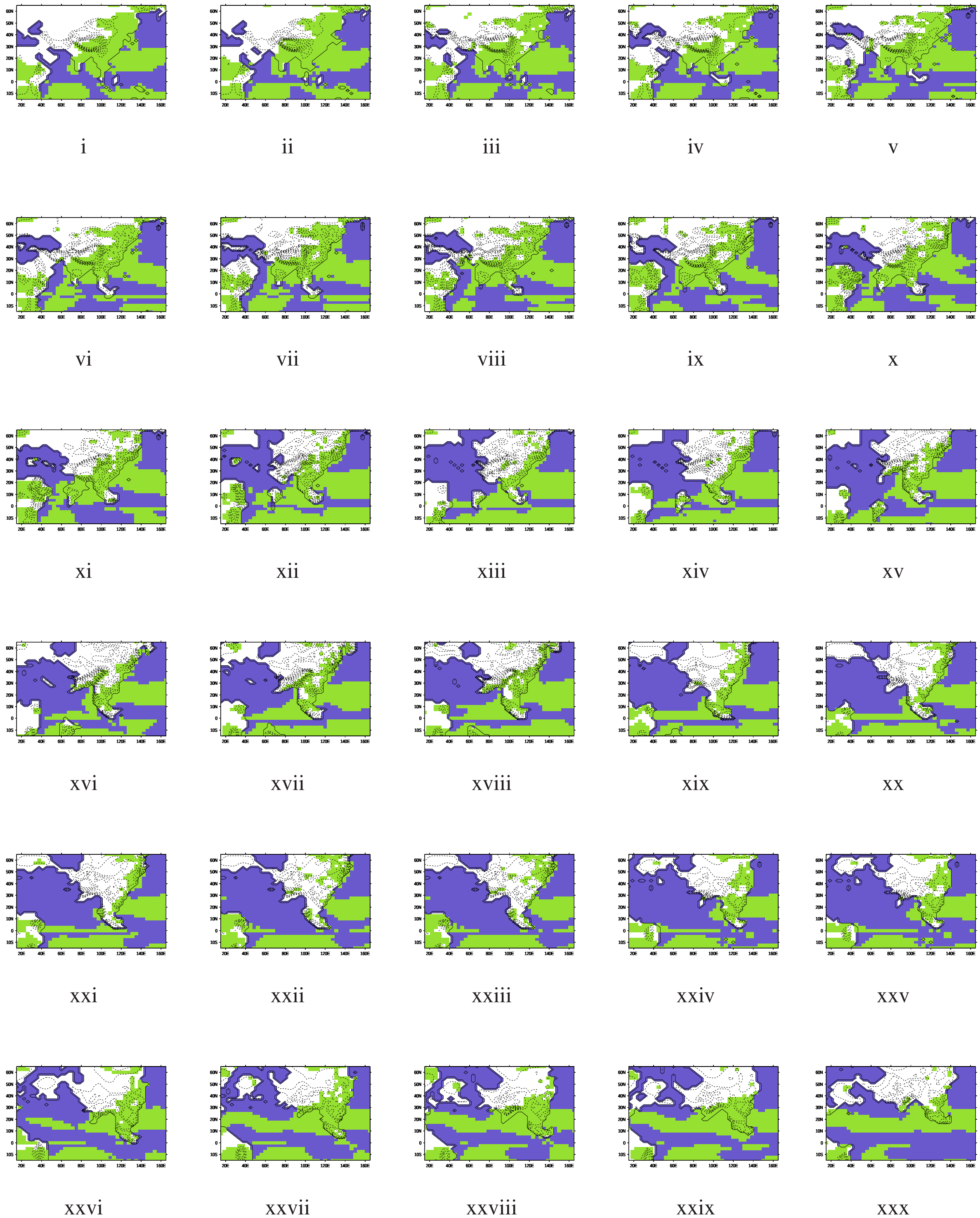

Figure S11 | Getech Palaeogeography Monsoon Domain (green area; Wang and Ding 2008) in (i) Gelasian, (ii) Piacenzian, (iii) Zanclean, (iv) Messinian, (v) Tortonian, (vi) Serravallian, (vii) Langhian, (viii) Burdigalian, (ix) Aquitanian, (x) Chattian, (xi) Rupelian, (xii) Pribonian, (xiii) Bartonian, (xiv) Lutetian, (xv) Ypresian, (xvi) Thanetian, (xvii) Selandian, (xviii) Danian, (xix) Maastrichtian, (xx) Campanian, (xxi) Santonian, (xxii) Coniacian, (xxiii) Turonian, (xxiv) Cenomanian, (xxv) Albian, (xxvi) Aptian, (xxvii) Barremian, (xxviii) Hauterivian, (xxix) Valanginian, (xxx) Berriasian simulations.

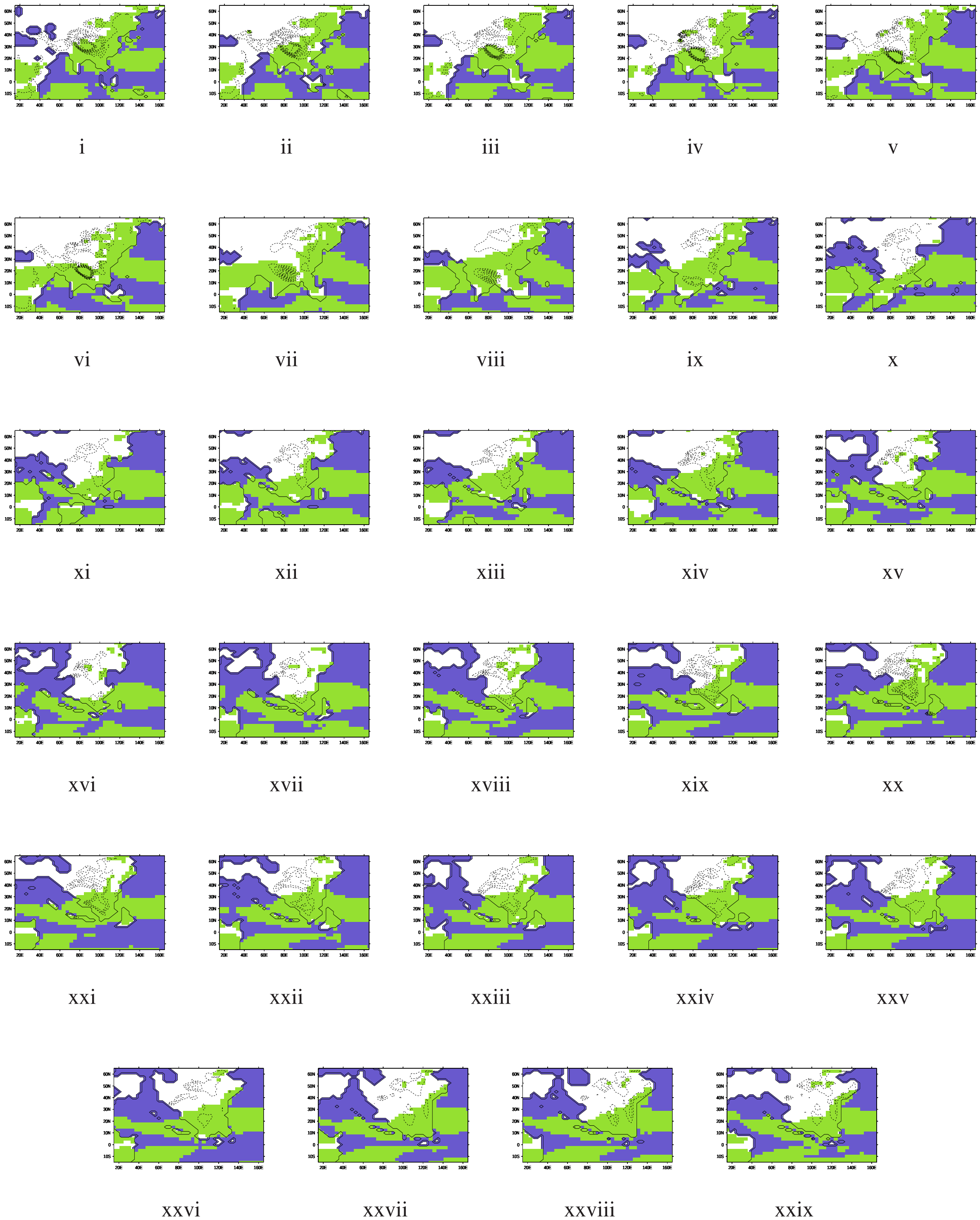

Figure S12 | Scotese Palaeogeography Monsoon Domain (green area; Wang and Ding 2008) in (i) Zanclean, (ii) Tortonian, (iii) Langhian, (iv) Burdigalian, (v) Chattian, (vi) Rupelian, (vii) Priabonian, (viii) Bartonian, (ix) Lutetian, (x) Ypresian, (xi) Thanetian, (xii) Selandian, (xiii) Danian, (xiv) Maastrictian, (xv) Late Campanian, (xvi) Early Campanian, (xvii) Santonian, (xviii) Turonian, (xix) Cenomanian, (xx) late Albian, (xxi) middle Albian, (xxii) early Albian, (xxiii) late Aptian, (xxiv) early Aptian, (xxv) Barremian, (xxvi) Hauterivian, (xxvii) Valanginian, (xxviii) late Barremian, (xxix) early Berriasian simulations.

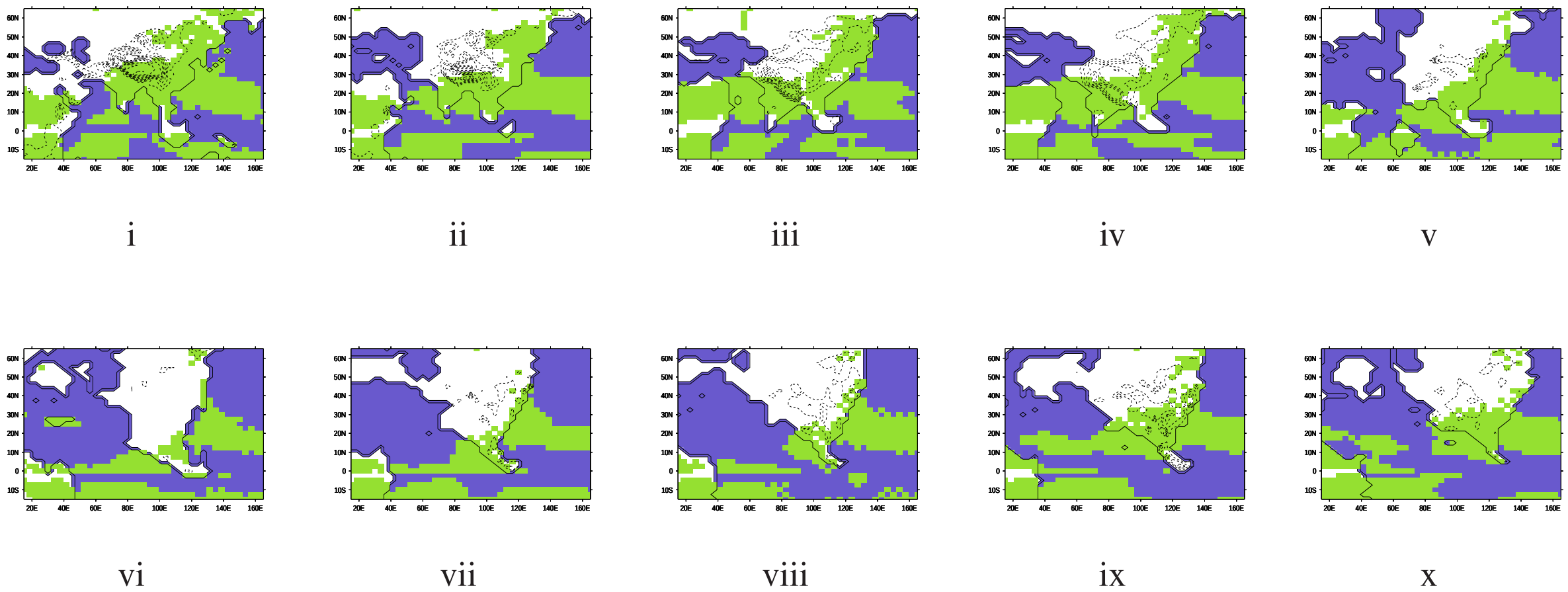

Figure S13 | Robertsons Palaeogeography Monsoon Domain (green area; Wang and Ding 2008) in (i) Piacenzian, (ii) Serravallian, (iii) Chattian, (iv) Rupelian, (v) Ypresian, (vi) Maastrichtian, (vii) Turonian, (viii) Albian, (ix) Aptian, (x) Berriasian simulations.

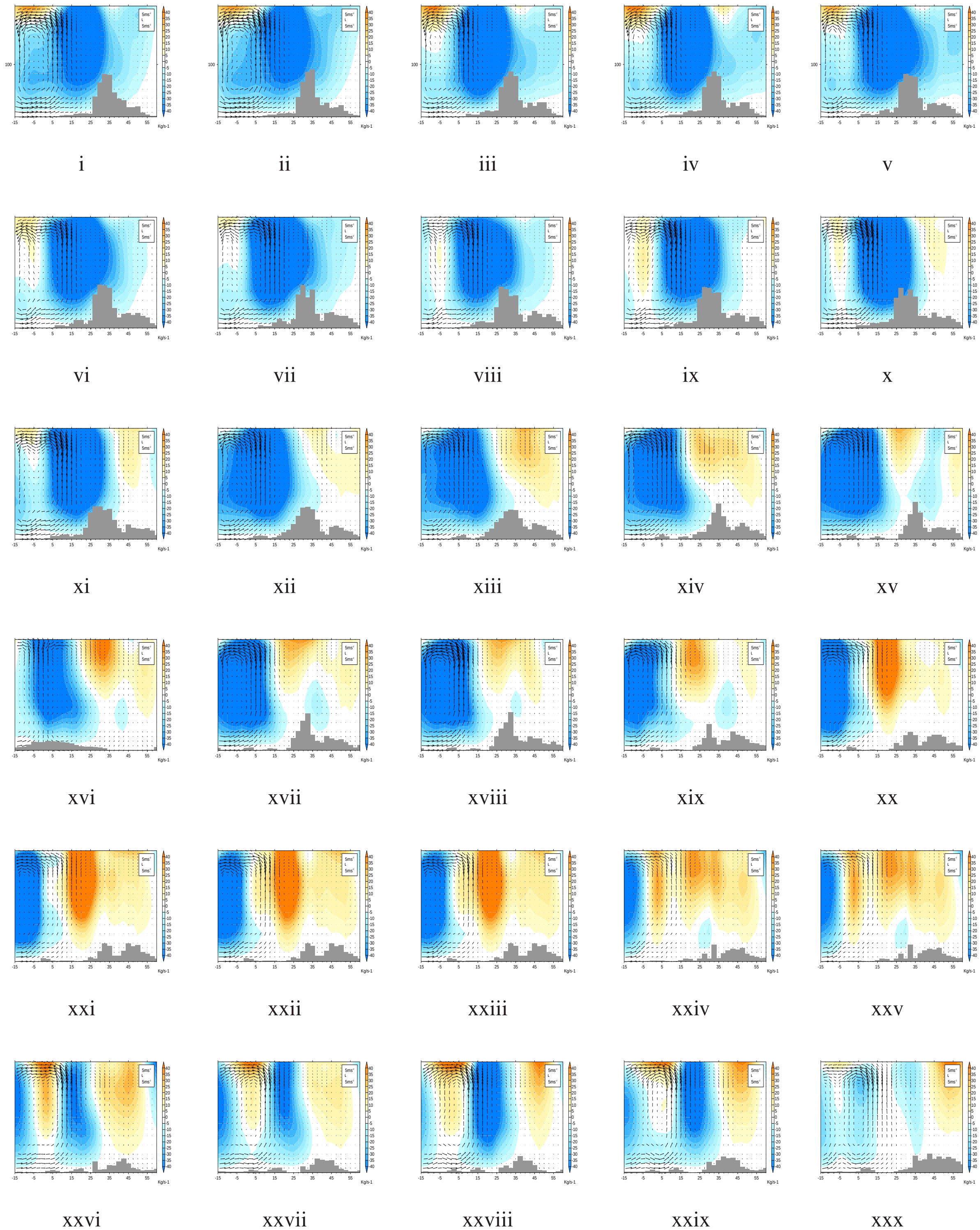

Figure S14 | Getech Palaeogeography Hadley circulation (atmospheric streamfunction) and vector winds (100E-120E) in (i) Gelasian, (ii) Piacenzian, (iii) Zanclean, (iv) Messinian, (v) Tortonian, (vi) Serravallian, (vii) Langhian, (viii) Burdigalian, (ix) Aquitanian, (x) Chattian, (xi) Rupelian, (xii) Pribonian, (xiii) Bartonian, (xiv) Lutetian, (xv) Ypresian, (xvi) Thanetian, (xvii) Selandian, (xviii) Danian, (xix) Maastrichtian, (xx) Campanian, (xxi) Santonian, (xxii) Coniacian, (xxiii) Turonian, (xxiv) Cenomanian, (xxv) Albian, (xxvi) Aptian, (xxvii) Barremian, (xxviii) Hauterivian, (xxix) Valanginian, (xxx) Berriasian simulations. Grey area represents mean TIbetan vertical profile between 75E-95E.

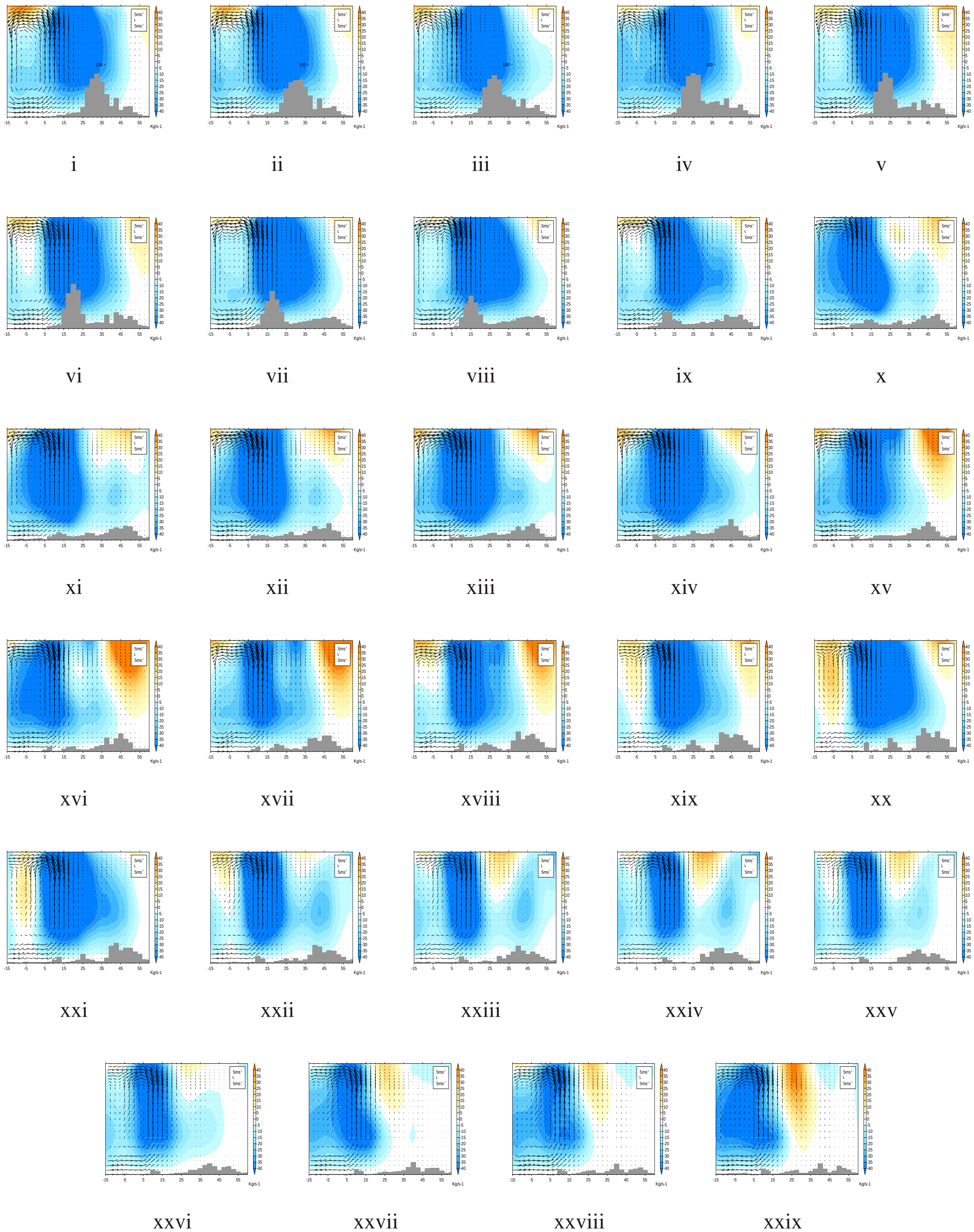

Figure S15 | Scotese Palaeogeography Hadley circulation (atmospheric streamfunction) and vector winds (100E-120E) in (i) Zanclean, (ii) Tortonian, (iii) Langhian, (iv) Burdigalian, (v) Chattian, (vi) Rupelian, (vii) Priabonian, (viii) Bartonian, (ix) Lutetian, (x) Ypresian, (xi) Thanetian, (xii) Selandian, (xiii) Danian, (xiv) Maastrictian, (xv) Late Campanian, (xvi) Early Campanian, (xvii) Santonian, (xviii) Turonian, (xix) Cenomanian, (xx) late Albian, (xxi) middle Albian, (xxii) early Albian, (xxiii) late Aptian, (xxiv) early Aptian, (xxv) Barremian, (xxvi) Hauterivian, (xxvii) Valanginian,(xxviii) late Barremian, (xxix) early Berriasian simulations. Grey area represents mean TIbetan vertical profile between 75E-95E.

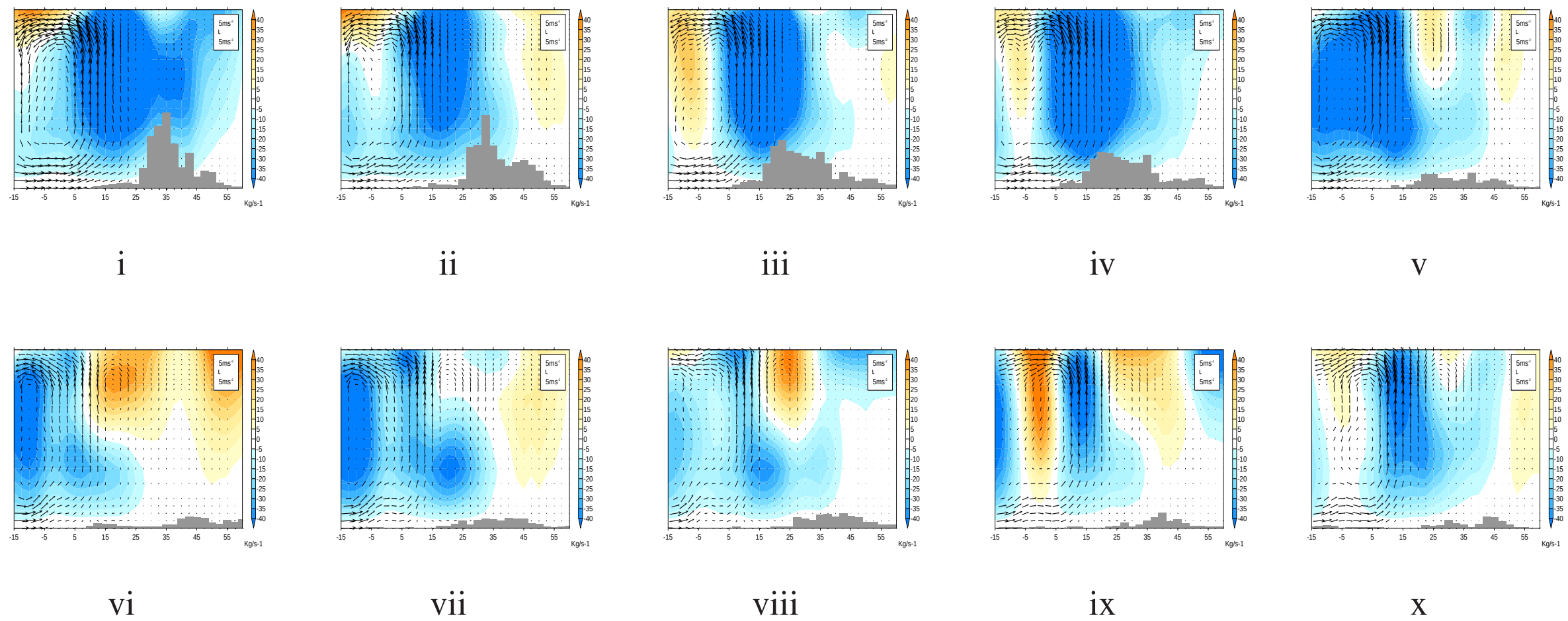

Figure S16 | Robertsons Palaeogeography Hadley circulation (atmospheric streamfunction) and vector winds (100E-120E) in (i) Piacenzian, (ii) Serravallian, (iii) Chattian, (iv) Rupelian, (v) Ypresian, (vi) Maastrichtian, (vii) Turonian, (viii) Albian, (ix) Aptian, (x) Berriasian simulations. Grey area represents mean TIbetan vertical profile between 75E-95E.

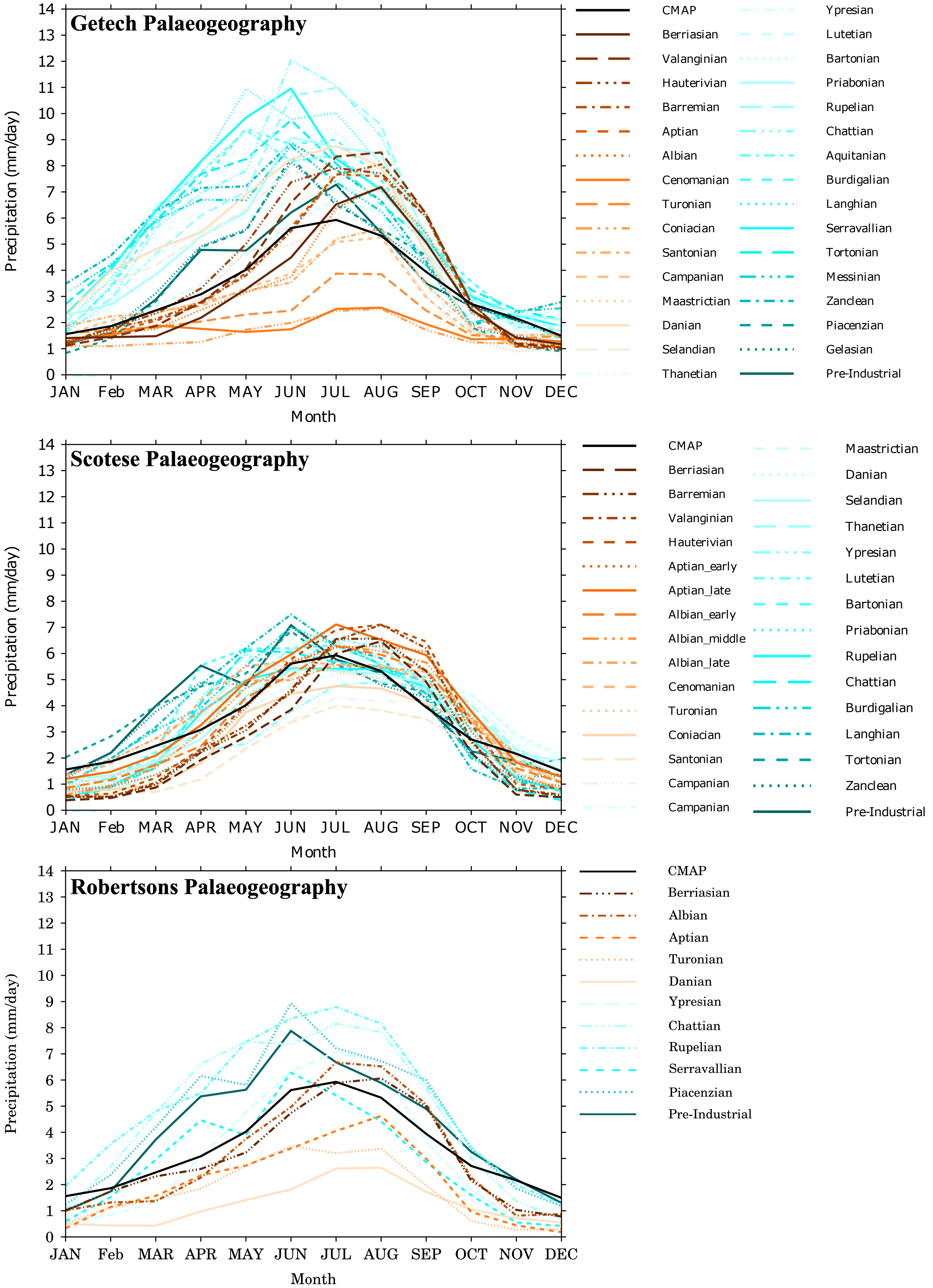

Figure S17 | Regional seasonal precipitation distribution. East Asian summer monsoon region seasonal precipitation (mm/day) timeseries for Getech and Robertsons palaeogeographies for each representative stages in each reconstruction.

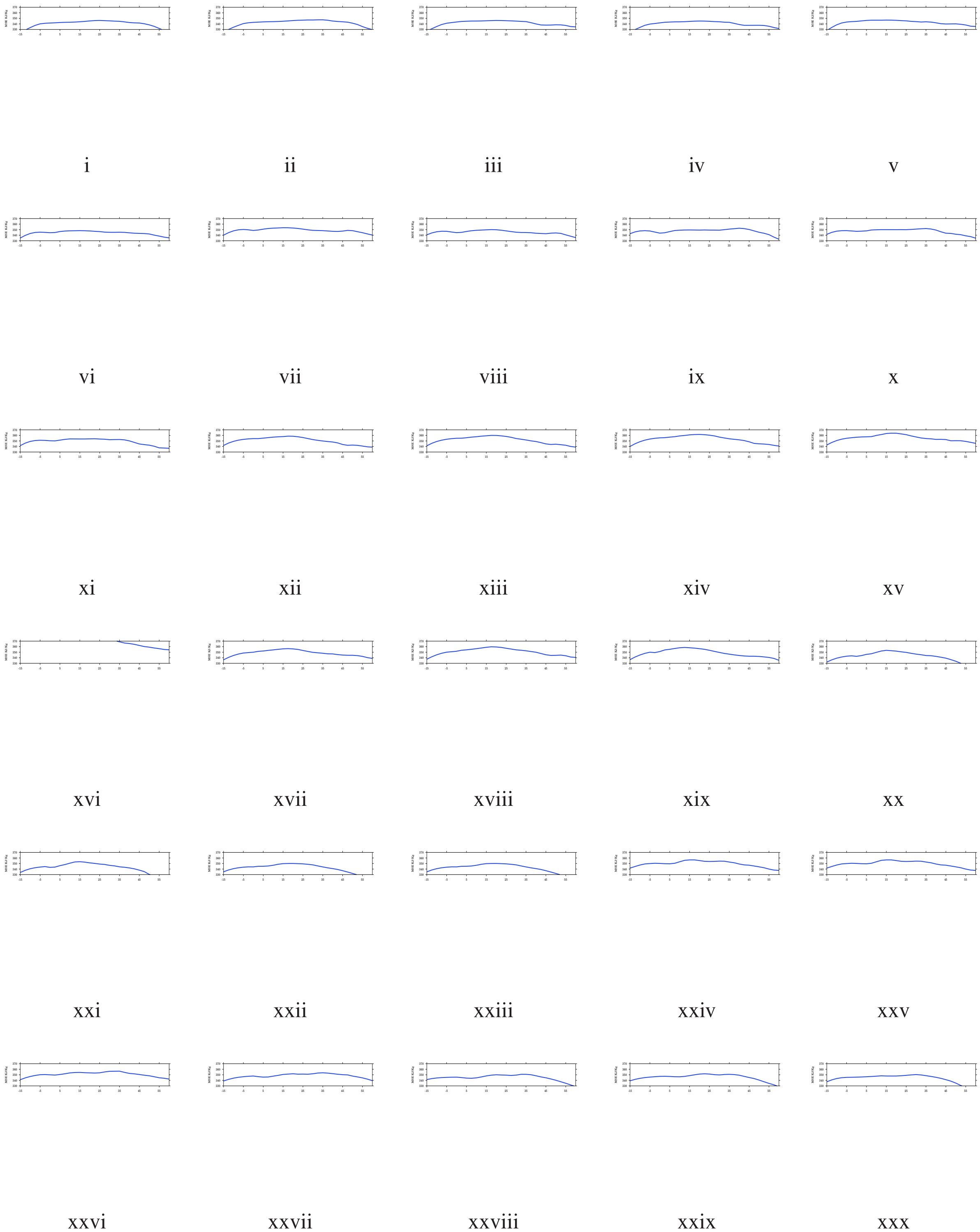

Figure S18 | Getech zonal mean poleward maxima subcloud moist static energy (KJ/Kg) bwtween 100E-120E in (i) Gelasian, (ii) Piacenzian, (iii) Zanclean, (iv) Messinian, (v) Tortonian, (vi) Serravallian, (vii) Langhian, (viii) Burdigalian, (ix) Aquitanian, (x) Chattian, (xi) Rupelian, (xii) Pribonian, (xiii) Bartonian, (xiv) Lutetian, (xv) Ypresian, (xvi) Thanetian, (xvii) Selandian, (xviii) Danian, (xix) Maastrichtian, (xx) Campanian, (xxi) Santonian, (xxii) Coniacian, (xxiii) Turonian, (xxiv) Cenomanian, (xxv) Albian, (xxvi) Aptian, (xxvii) Barremian, (xxviii) Hauterivian, (xxix) Valanginian, (xxx) Berriasian simulations.

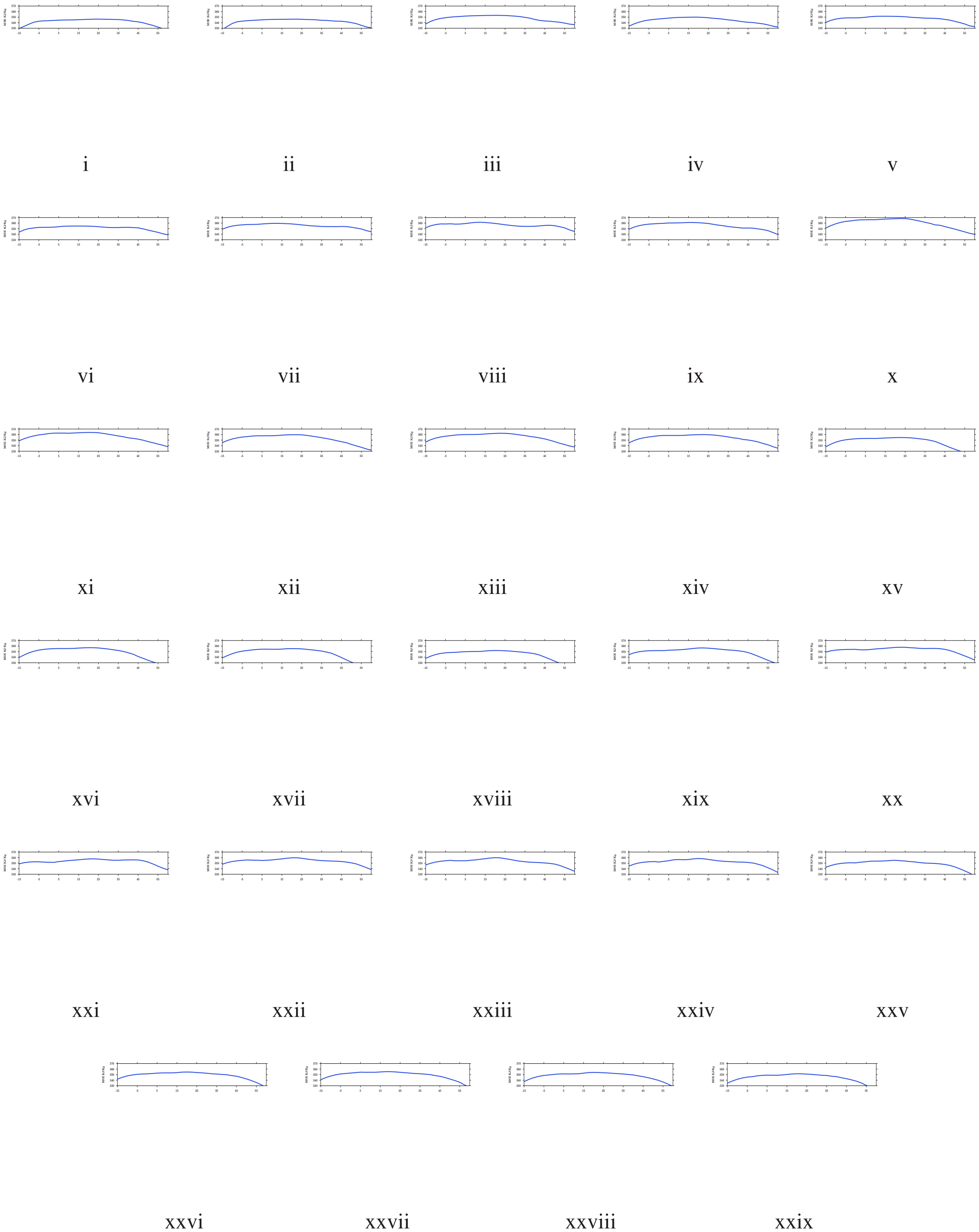

Figure S19 | Scotese zonal mean subcloud moist static energy (KJ/Kg) bwtween 100E-120E in (i) Zanclean, (ii) Tortonian, (iii) Langhian, (iv) Burdigalian, (v) Chattian, (vi) Rupelian, (vii) Priabonian, (viii) Bartonian, (ix) Lutetian, (x) Ypresian, (xi) Thanetian, (xii) Selandian, (xiii) Danian, (xiv) Maastrictian, (xv) Late Campanian, (xvi) Early Campanian, (xvii) Santonian, (xviii) Turonian, (xix) Cenomanian, (xx) late Albian, (xxi) middle Albian, (xxii) early Albian, (xxiii) late Aptian, (xxiv) early Aptian, (xxv) Barremian, (xxvi) Hauterivian, (xxvii) Valanginian, (xxviii) late Barremian, (xxix) early Berriasian simulations.

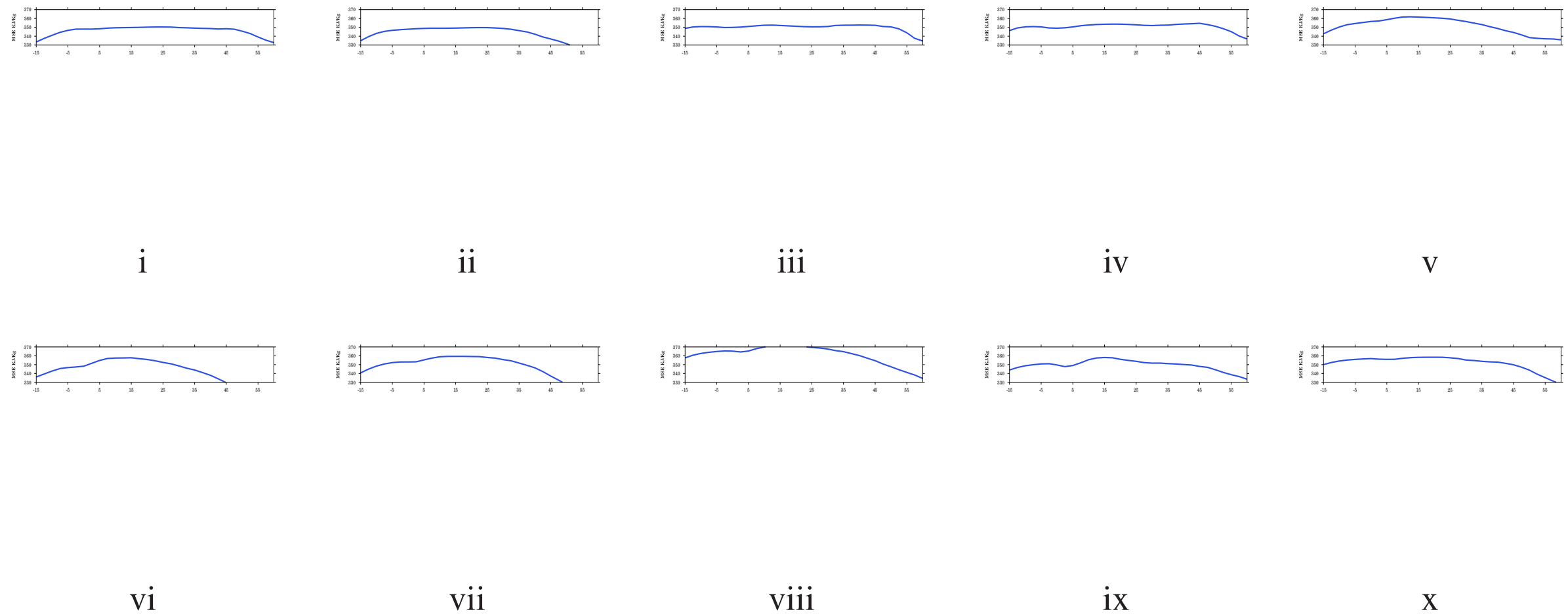

Figure S20 | Robertsons zonal mean subcloud moist static energy (KJ/Kg) bwtween 100E-120E in (i) Piacenzian, (ii) Serravallian, (iii) Chattian, (iv) Rupelian, (v) Ypresian, (vi) Maastrichtian, (vii) Turonian, (viii) Albian, (ix) Aptian, (x) Berriasian simulations.

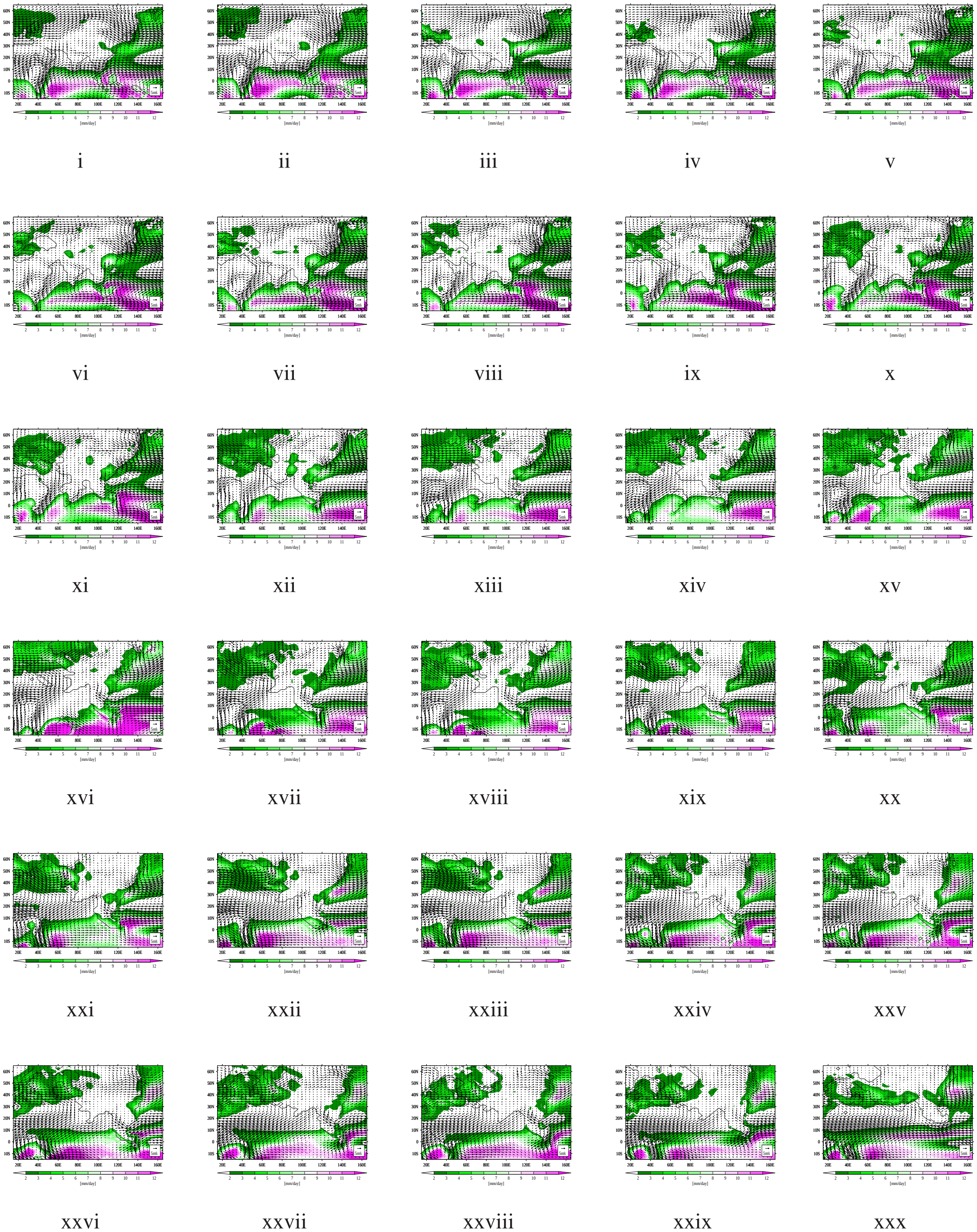

Figure S21 | Getech Palaeogeography DJF precipitation (mm/day) and 850 mb wind (m/s) in (i) Gelasian, (ii) Piacenzian, (iii) Zanclean, (iv) Messinian, (v) Tortonian, (vi) Serravallian, (vii) Langhian, (viii) Burdigalian, (ix) Aquitanian, (x) Chattian, (xi) Rupelian, (xii) Pribonian, (xiii) Bartonian, (xiv) Lutetian, (xv) Ypresian, (xvi) Thanetian, (xvii) Selandian, (xviii) Danian, (xix) Maastrichtian, (xx) Campanian, (xxi) Santonian, (xxii) Coniacian, (xxiii) Turonian, (xxiv) Cenomanian, (xxv) Albian, (xxvi) Aptian, (xxvii) Barremian, (xxviii) Hauterivian, (xxix) Valanginian, (xxx) Berriasian simulations.

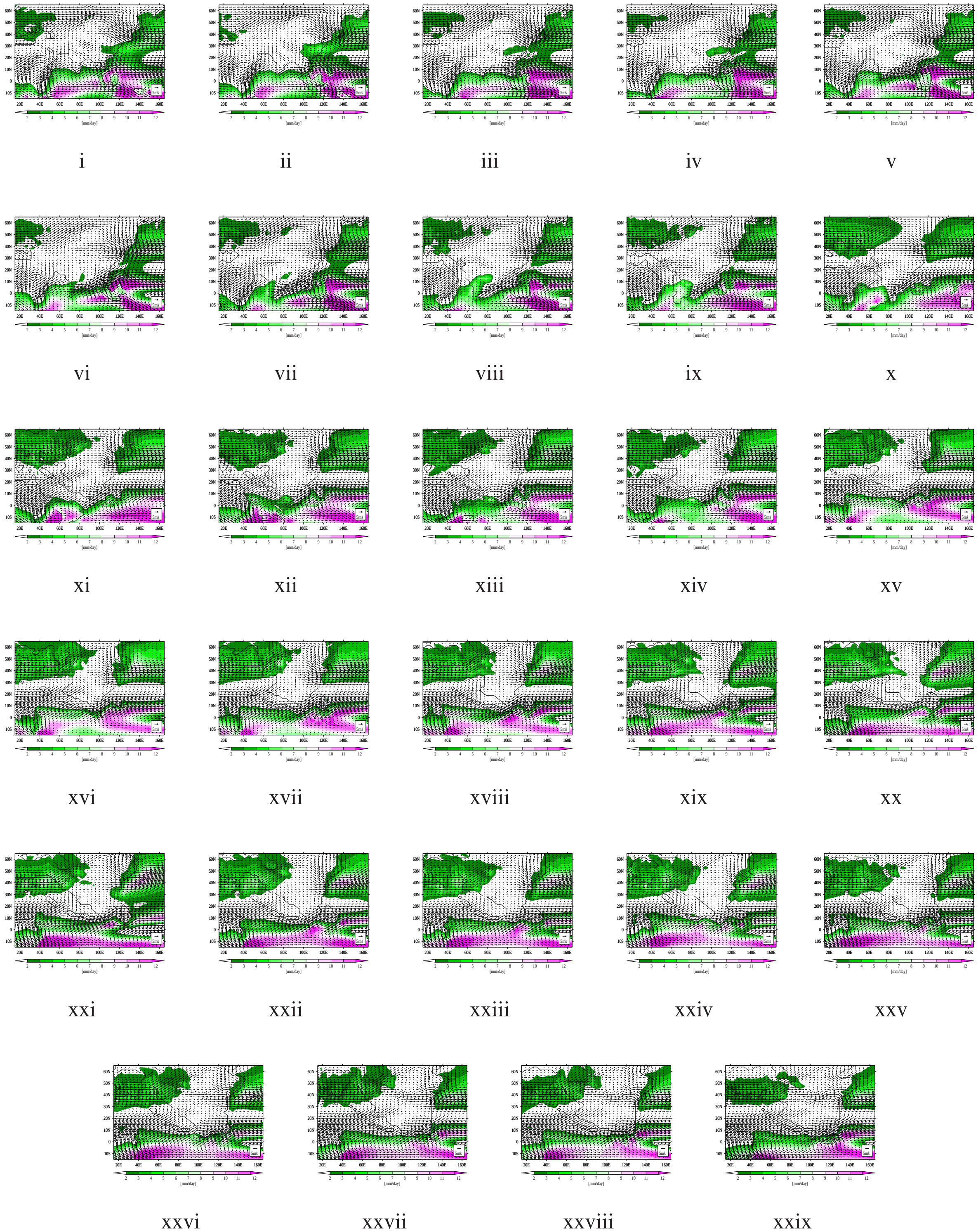

Figure S22 | Scotese Palaeogeography DJF precipitation (mm/day) and 850 mb wind (m/s) in (i) Zanclean, (ii) Tortonian, (iii) Langhian, (iv) Burdigalian, (v) Chattian, (vi) Rupelian, (vii) Priabonian, (viii) Bartonian, (ix) Lutetian, (x) Ypresian, (xi) Thanetian, (xii) Selandian, (xiii) Danian, (xiv) Maastrictian, (xv) Late Campanian, (xvi) Early Campanian, (xvii) Santonian, (xviii) Turonian, (xix) Cenomanian, (xx) late Albian, (xxi) middle Albian, (xxii) early Albian, (xxiii) late Aptian, (xxiv) early Aptian, (xxv) Barremian, (xxvi) Hauterivian, (xxvii) Valanginian, (xxviii) late Barremian, (xxix) early Berriasian simulations.

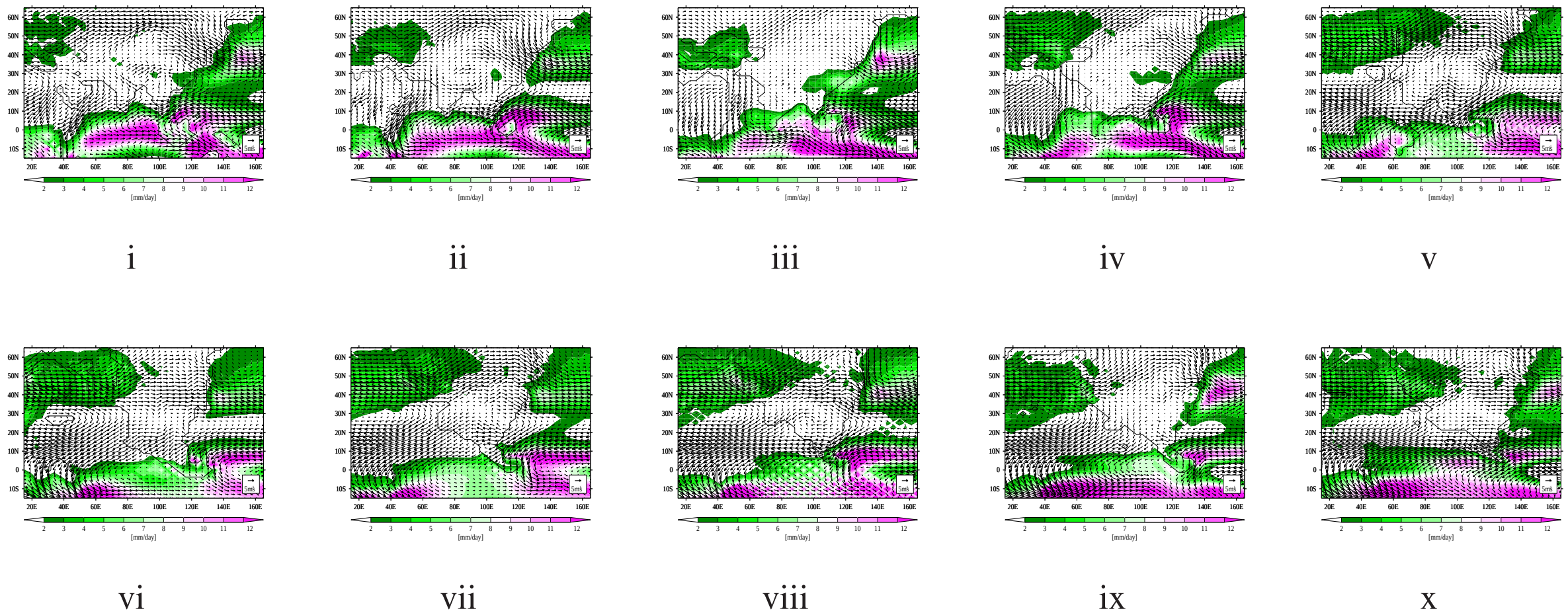

Figure S23 | Robertsons Palaeogeography DJF Precipitation (mm/day) and and 850 mb wind (m/s) in (i) Piacenzian, (ii) Serravallian, (iii) Chattian, (iv) Rupelian, (v) Ypresian, (vi) Maastrichtian, (vii) Turonian, (viii) Albian, (ix) Aptian, (x) Berriasian simulations.

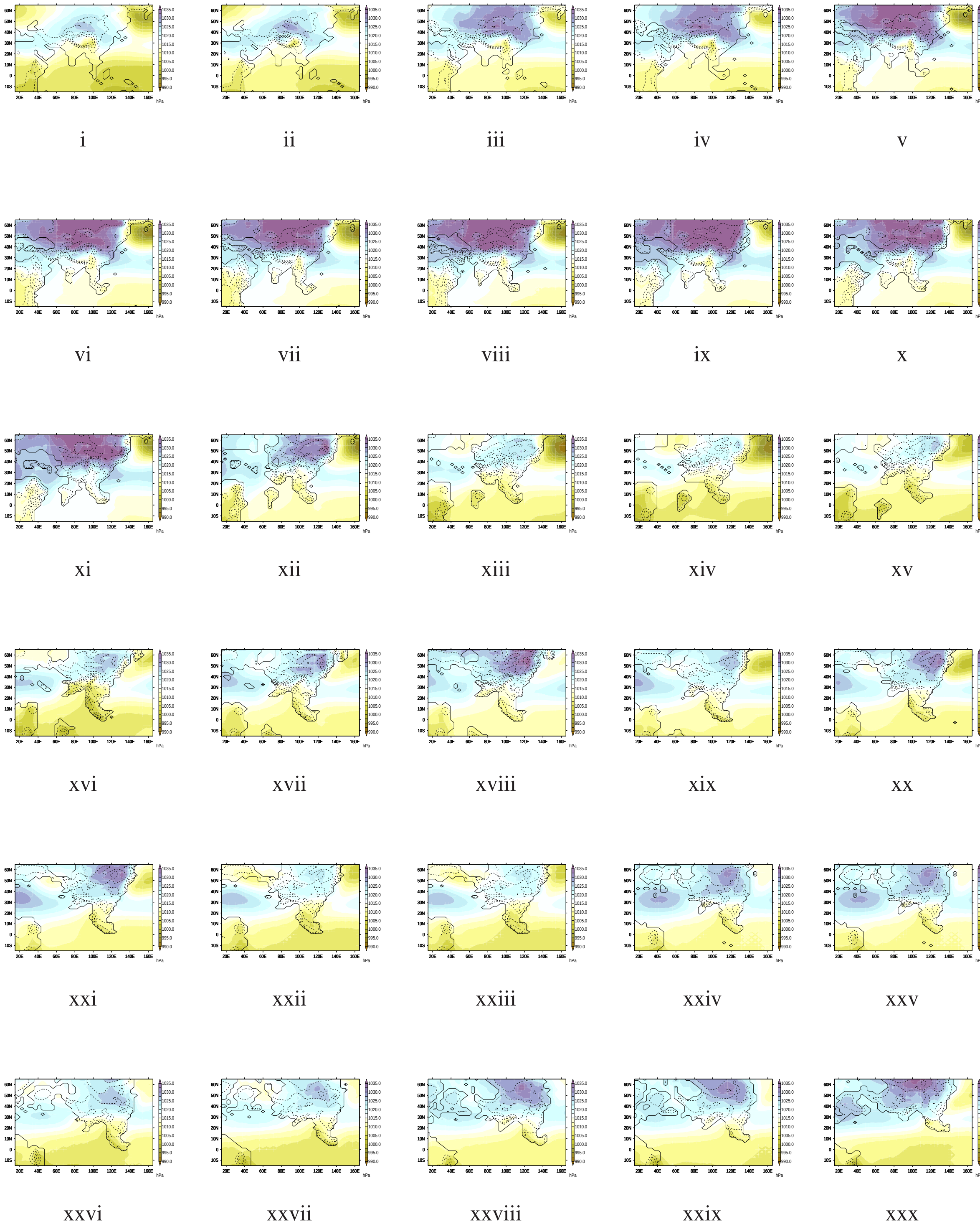

Figure S24 | Getech Palaeogeography DJF mean sea level pressure (mslp; hPa) in (i) Gelasian, (ii) Piacenzian, (iii) Zanclean, (iv) Messinian, (v) Tortonian, (vi) Serravallian, (vii) Langhian, (viii) Burdigalian, (ix) Aquitanian, (x) Chattian, (xi) Rupelian, (xii) Pribonian, (xiii) Bartonian, (xiv) Lutetian, (xv) Ypresian, (xvi) Thanetian, (xvii) Selandian, (xviii) Danian, (xix) Maastrichtian, (xx) Campanian, (xxi) Santonian, (xxii) Coniacian, (xxiii) Turonian, (xxiv) Cenomanian, (xxv) Albian, (xxvi) Aptian, (xxvii) Barremian, (xxviii) Hauterivian, (xxix) Valanginian, (xxx) Berriasian simulations. Dashed lines represent 1000m topography contour.

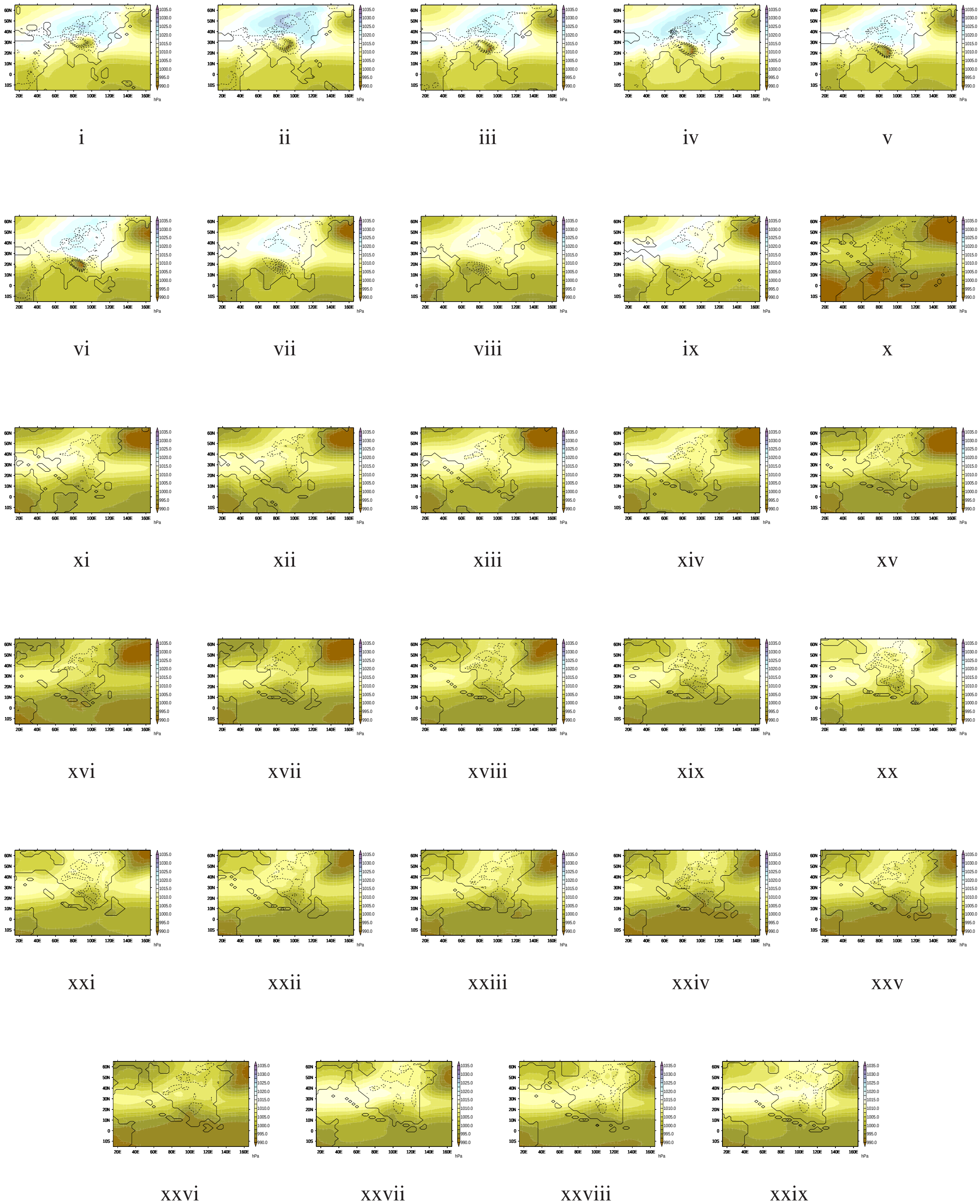

Figure S25 | Scotese Palaeogeography DJF mean sea level pressure (mslp; hPa) in (i) Zanclean, (ii) Tortonian, (iii) Langhian, (iv) Burdigalian, (v) Chattian, (vi) Rupelian, (vii) Priabonian, (viii) Bartonian, (ix) Lutetian, (x) Ypresian, (xi) Thanetian, (xii) Selandian, (xiii) Danian, (xiv) Maastrictian, (xv) Late Campanian, (xvi) Early Campanian, (xvii) Santonian, (xviii) Turonian, (xix) Cenomanian, (xx) late Albian, (xxi) middle Albian, (xxii) early Albian, (xxiii) late Aptian, (xxiv) early Aptian, (xxv) Barremian, (xxvi) Hauterivian, (xxvii) Valanginian, (xxviii) late Barremian, (xxix) early Berriasian simulations. Dashed lines represent 1000m topography contour.

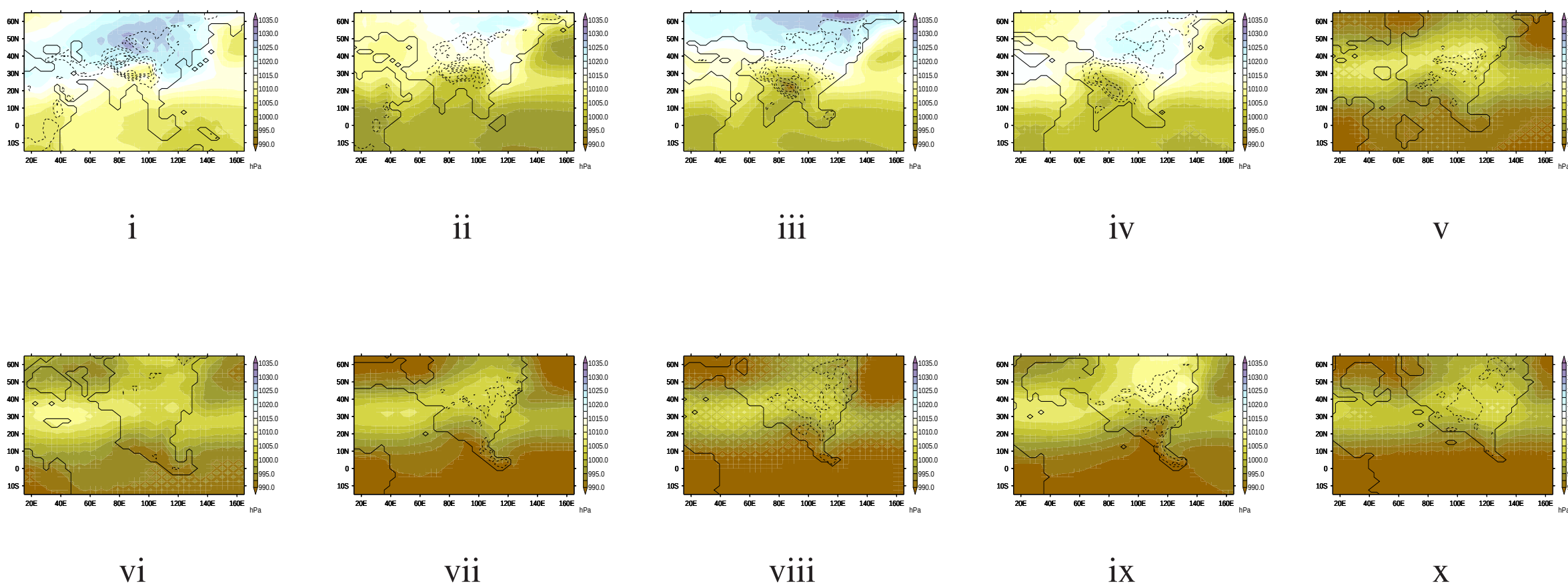

Figure S26 | Robertsons Palaeogeography DJF mean sea level pressure (mslp; hPa) in (i) Piacenzian, (ii) Serravallian, (iii) Chattian, (iv) Rupelian, (v) Ypresian, (vi) Maastrichtian, (vii) Turonian, (viii) Albian, (ix) Aptian, (x) Berriasian simulations. Dashed lines represent 1000m topography contour.

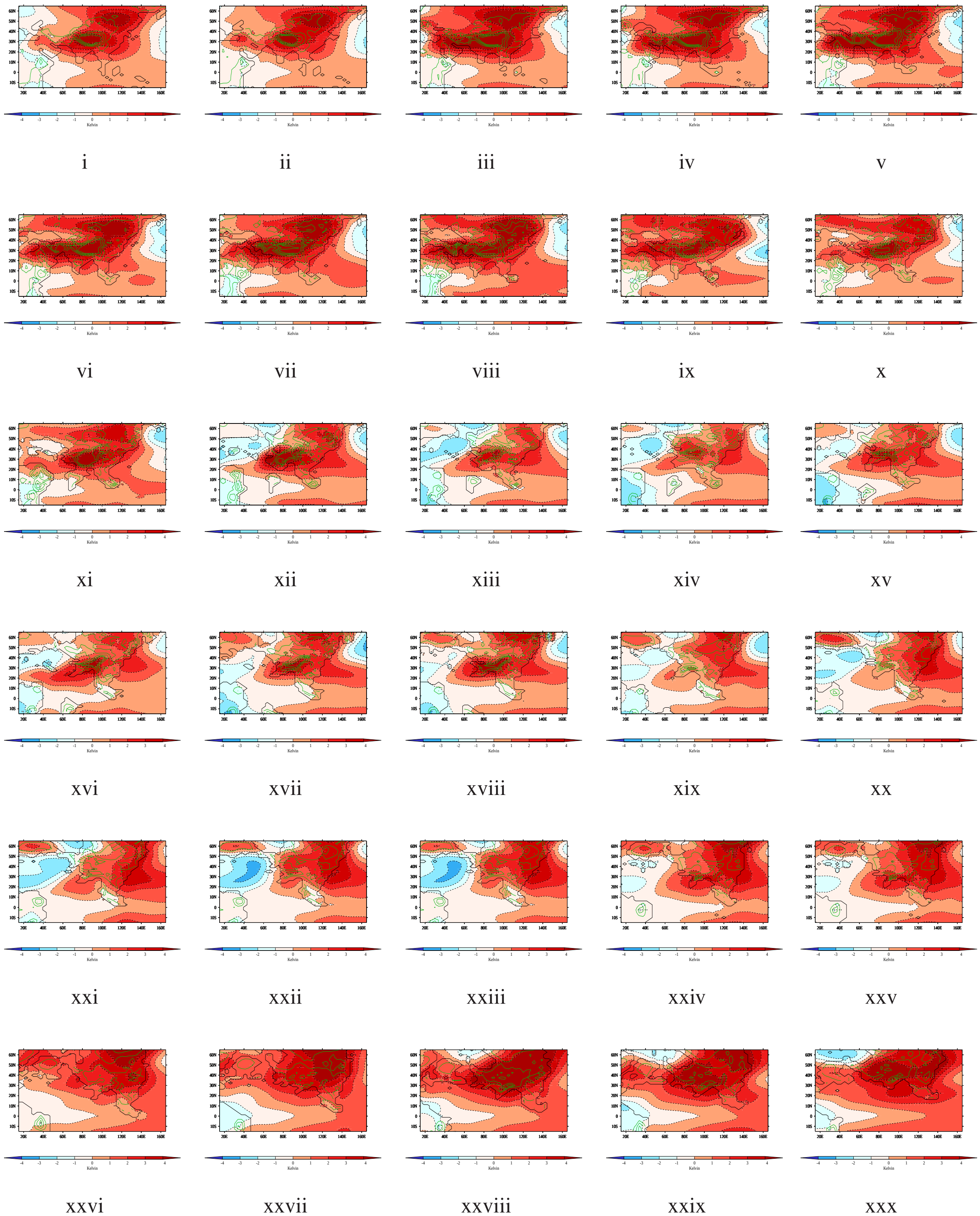

Figure S27 | Getech Palaeogeography JJA 500 hPa temperature anomaly (zonal mean removed) in (i) Gelasian, (ii) Piacenzian, (iii) Zanclean, (iv) Messinian, (v) Tortonian, (vi) Serravallian, (vii) Langhian, (viii) Burdigalian, (ix) Aquitanian, (x) Chattian, (xi) Rupelian, (xii) Pribonian, (xiii) Bartonian, (xiv) Lutetian, (xv) Ypresian, (xvi) Thanetian, (xvii) Selandian, (xviii) Danian, (xix) Maastrichtian, (xx) Campanian, (xxi) Santonian, (xxii) Coniacian, (xxiii) Turonian, (xxiv) Cenomanian, (xxv) Albian, (xxvi) Aptian, (xxvii) Barremian, (xxviii) Hauterivian, (xxix) Valanginian, (xxx) Berriasian simulations. Green lines represent 1000m topography contour.

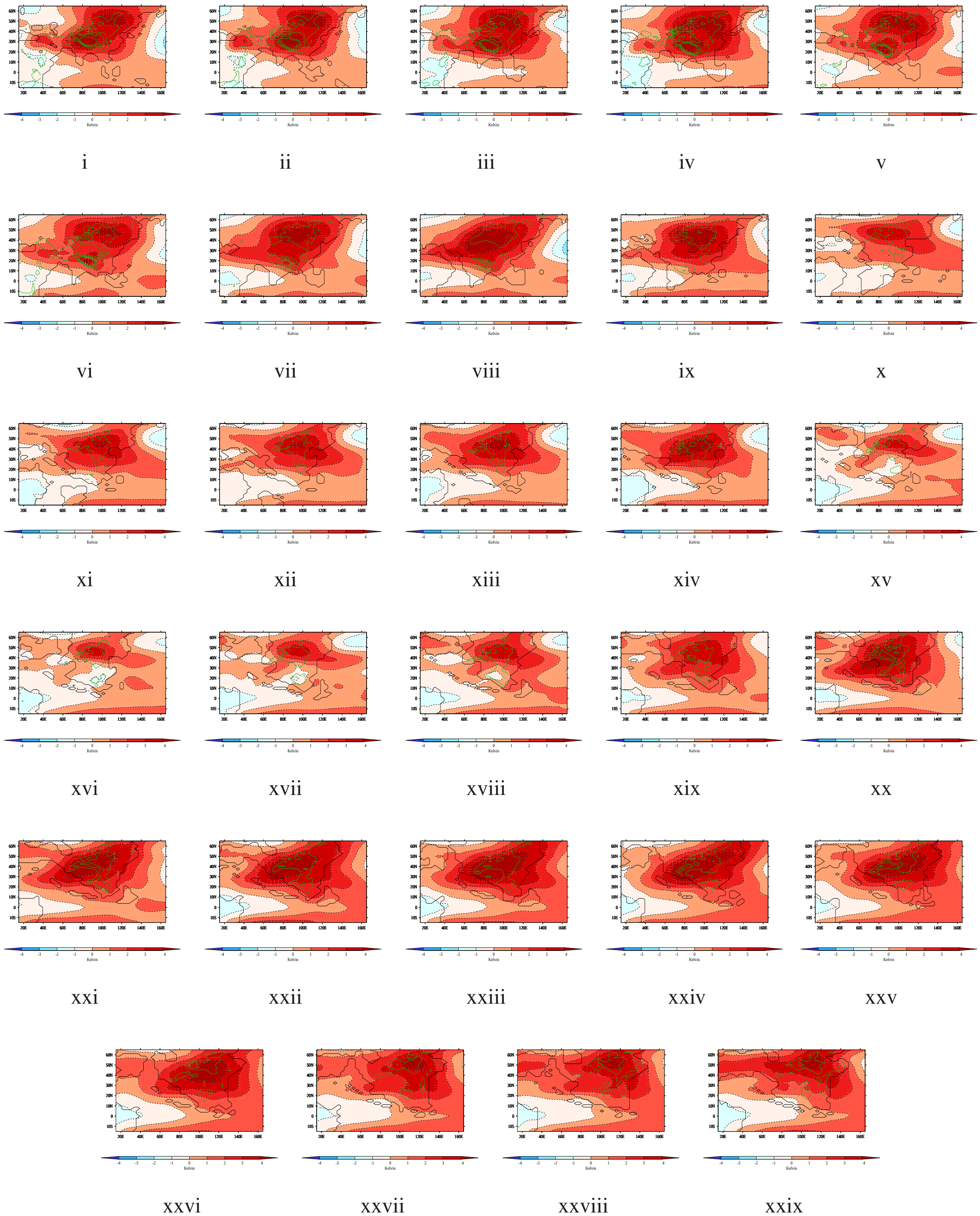

Figure S28 | Scotese Palaeogeography JJA 500 hPa temperature anomaly (zonal mean removed) in (i) Zanclean, (ii) Tortonian, (iii) Langhian, (iv) Burdigalian, (v) Chattian, (vi) Rupelian, (vii) Priabonian, (viii) Bartonian, (ix) Lutetian, (x) Ypresian, (xi) Thanetian, (xii) Selandian, (xiii) Danian, (xiv) Maastrictian, (xv) Late Campanian, (xvi) Early Campanian, (xvii) Santonian, (xviii) Turonian, (xix) Cenomanian, (xx) late Albian, (xxi) middle Albian, (xxii) early Albian, (xxiii) late Aptian, (xxiv) early Aptian, (xxv) Barremian, (xxvi) Hauterivian, (xxvii) Valanginian, (xxviii) late Barremian, (xxix) early Berriasian simulations. Green lines represent 1000m topography contour.

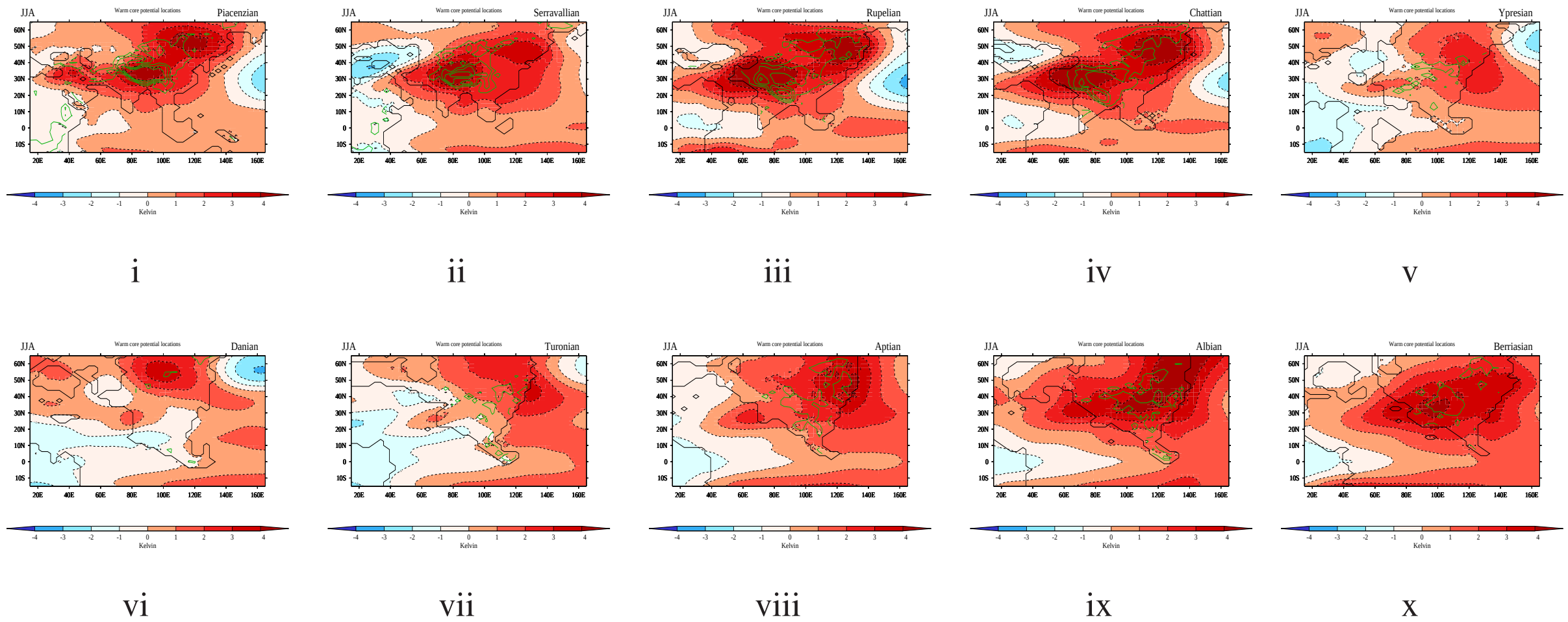

Figure S29 | Robertsons Palaeogeography JJA 500 hPa temperature anomaly (zonal mean removed) in (i) Piacenzian, (ii) Serravallian, (iii) Chattian, (iv) Rupelian, (v) Ypresian, (vi) Maastrichtian, (vii) Turonian, (viii) Albian, (ix) Aptian, (x) Berriasian simulations. Green lines represent 1000m topography contour.

References:

1 Valdes, P. J. *et al.* The BRIDGE HadCM3 family of climate models: HadCM3@Bristol v1.0. *Geosci Model Dev* **10**, 3715-3743 (2017). https://doi.org:10.5194/gmd-10-3715-2017

2 Cox, P. M. Description of the TRIFFID dynamic global vegetation

model, Tech. rep., Met Office Hadley Centre, Exeter, UK. (2000).

3 Cox, P. M. Aprimitive equation, 3-dimensional model of the ocean, GFDL Ocean Group Technical Report No. 1. *MetOffice technical report* (1984).

4 Collins, M., Tett, S. F. B. & Cooper, C. The internal climate variability of HadCM3, a version of the Hadley Centre coupled model without flux adjustments. *Clim Dynam* **17**, 61-81 (2001). https://doi.org:DOI 10.1007/s003820000094

5 Sagoo, N., Valdes, P., Flecker, R. & Gregoire, L. J. The Early Eocene equable climate problem: can perturbations of climate model parameters identify possible solutions? *Philosophical Transactions of the Royal Society A: Mathematical, Physical and Engineering Sciences* **371**, 20130123 (2013). https://doi.org:doi:10.1098/rsta.2013.0123

6 Kiehl, J. T. & Shields, C. A. Sensitivity of the Palaeocene–Eocene Thermal Maximum climate to cloud properties. *Philosophical Transactions of the Royal Society A: Mathematical, Physical and Engineering Sciences* **371**, 20130093 (2013). https://doi.org:doi:10.1098/rsta.2013.0093

7 Sperber, K. *et al.* The Asian summer monsoon: an intercomparison of CMIP5 vs. CMIP3 simulations of the late 20th century. *Clim Dynam* **41**, 2711-2744 (2013). https://doi.org:10.1007/s00382-012-1607-6

8 Farnsworth, A. *et al.* Past East Asian monsoon evolution controlled by paleogeography, not CO2. *Sci Adv* **5** (2019). https://doi.org:ARTN eaax1697

10.1126/sciadv.aax1697

9 Jiang, D. B., Wang, H. J. & Lang, X. M. Evaluation of East Asian climatology as simulated by seven coupled models. *Adv Atmos Sci* **22**, 479-495 (2005).

10 Schneider, U. *et al.* GPCC's new land surface precipitation climatology based on quality-controlled in situ data and its role in quantifying the global water cycle. *Theor Appl Climatol* **115**, 15-40 (2014). https://doi.org:10.1007/s00704-013-0860-x

11 Scotese, C. R. PALEOMAP Project (2007). (2007).

12 Rae, J. W. B. *et al.* Atmospheric CO2 over the Past 66 Million Years from Marine Archives. *Annual Review of Earth and Planetary Sciences* **49**, 609-641 (2021). https://doi.org:https://doi.org/10.1146/annurev-earth-082420-063026

13 Foster, G. L., Royer, D. L. & Lunt, D. J. Future climate forcing potentially without precedent in the last 420 million years. *Nat Commun* **8** (2017). https://doi.org:ARTN 14845

10.1038/ncomms14845

14 Gough, D. O. Solar Interior Structure and Luminosity Variations. *Sol Phys* **74**, 21-34 (1981). https://doi.org:Doi 10.1007/Bf00151270

15 Beck, H. E. *et al.* Present and future Köppen-Geiger climate classification maps at 1-km resolution. *Scientific Data* **5**, 180214 (2018). https://doi.org:10.1038/sdata.2018.214
